# Cancer cell responses to Hsp70 inhibitor JG-98: Comparison with Hsp90 inhibitors and finding synergistic drug combinations

**DOI:** 10.1038/s41598-017-14900-0

**Published:** 2018-02-14

**Authors:** Julia A. Yaglom, Yongmei Wang, Amy Li, Zhenghu Li, Stephano Monti, Ilya Alexandrov, Xiongbin Lu, Michael Y. Sherman

**Affiliations:** 1 Boston University School of Medicine, Boston, United States; 2 Center of Diagnosis and Treatment of Breast Disease, The Affiliated Hospital of Qingdao University, Qingdao, China; 3 MD Anderson Cancer Center, Houston, USA; 4 ActivSignal, Watertown, MA USA; 5 Department Molecular Biology Ariel University, Ariel, Israel

## Abstract

Hsp70 is a promising anti-cancer target. Our JG-98 series of Hsp70 inhibitors show anti-cancer activities affecting both cancer cells and tumor-associated macrophages. They disrupt Hsp70 interaction with a co-chaperone Bag3 and affect signaling pathways important for cancer development. Due to a prior report that depletion of Hsp70 causes similar responses as depletion of Hsp90, interest to Hsp70 inhibitors as drug prototypes is hampered by potential similarity of their effects to effects of Hsp90 inhibitors. Here, using the Connectivity Map platform we demonstrate that physiological effects of JG-98 are dissimilar from effects of Hsp90 inhibitors, thus justifying development of these compounds. Using gene expression and ActivSignal IPAD platform, we identified pathways modulated by JG-98. Some of these pathways were affected by JG-98 in Bag3-dependent (e.g. ERK) and some in Bag3-independent manner (e.g. Akt or c-myc), indicating multiple effects of Hsp70 inhibition. Further, we identified genes that modulate cellular responses to JG-98, developed approaches to predict potent combinations of JG-98 with known drugs, and demonstrated that inhibitors of proteasome, RNApol, Akt and RTK synergize with JG-98. Overall, here we established unique effects of novel Hsp70 inhibitors on cancer cell physiology, and predicted potential drug combinations for pre-clinical development.

## Introduction

The major molecular chaperone Hsp70 (HspA1A)^1–3^ has been implicated in cancer. Hsp70 levels are highly elevated in a variety of tumors^4–7^, and expression of Hsp70 strongly correlates with tumor grade, metastasis, and poor prognosis, suggesting that it plays a special and broad role in cancer^8^. Indeed, genetic ablation of Hsp70 suppresses cancer development in mouse models^9–11^. Furthermore, Hsp70 is involved in cancer development at multiple steps, both in initiation and progression.

While Hsp70 is critical for proliferation of cancer cells, it is dispensable for growth of non-transformed cells^9^, and the Hsp70 knockout mouse is healthy^12^ exhibiting only sensitivity to stress^12,13^, or acute inflammation^14^. The specific requirements for Hsp70 for cancer development suggest that this protein could be used as a drug target, and a number of small molecules that target Hsp70 have been developed^15–21^. The concern with this approach is that Hsp70 is involved in many normal pathways, so the safety of its inhibition remains uncertain.

Using genetic models, we recently made an important discovery that some cancer-specific effects of Hsp70 result from its direct interaction with a co-chaperone Bag3, rather than from its core chaperone function. Hsp70-Bag3 module controls multiple signaling pathways that regulate cancer, suggesting that the Hsp70-Bag3 interaction, rather than Hsp70 chaperone activity, may be a safer and more effective target. Dr. Jason Gestwicki and his colleagues identified an allosteric inhibitor that binds Hsp70 and inhibits the Hsp70-Bag3 interaction^22^. A first generation molecule from this series, YM-01, mimics the effects of Hsp70 depletion on cancer signaling pathways and tumor growth^23^. From a hit-to-lead campaign, a second generation molecule JG-98 was identified with greater affinity for Hsp70, and stronger anti-cancer effects in animal models^24^. An unusual benefit of these inhibitors is that they not only reduce viability of cancer cells, but also reduce infiltration of tumor-associate macrophages^25^. Accordingly, by acting through the tumor stroma, JG-98 shows potent anti-cancer effects even if tumor is formed by cancer cells resistant to it^25^. Though these inhibitors do not distinguish between highly homologous Hsp70 family members, focusing on cancer specific functions addresses the daunting challenge of safety of Hsp70 inhibitors. Furthermore, several distinct members of Hsp70 family, e.g. mortalin^26,27^ or Grp78^28,29^ were also implicated in cancer, and targeting these proteins by JG-98 may provide additional benefits. Here we undertook a series of tests in order to uncover pathways regulated by JG-98-mediated inhibition of Hsp70 family members in cancer cells. These tests were followed by genetic analysis to dissect physiological significance of the pathways in response of cancer cells to JG-98 series of Hsp70 inhibitors. Finally, we used this information to identify known drugs that show synergistic effects on cancer cell killing when combined with JG-98.

## Results

### Physiological effects of JG-98 are distinct from those of Hsp90 inhibitors

Simultaneous depletion of two major Hsp70 family members, inducible Hsp70 and constitutive Hsc73, was reported to cause similar physiological responses as inhibition of Hsp90^30^. This report raises a question regarding the rational for development of novel Hsp70 inhibitors since very selective and potent Hsp90 inhibitors have already been developed, and demonstrated a number of drawbacks related to insufficient efficacy. Accordingly, recently developed Hsp70 inhibitors of JG-98 series could cause similar physiological responses as inhibitors of Hsp90, significantly reducing potential importance of development of these novel compounds as drugs. To address this question, we sought to compare physiological effects of inhibitors of Hsp90 and JG-98 series of Hsp70 inhibitors. For that purpose, we employed the Broad Institute platform L1000 to compare limited gene expression patterns in multiple cell lines following treatment with either JG-98 or Hsp90 inhibitor, tanespimycin. L1000 platform generates signatures of perturbations (~20000 compounds) in multiple cell lines. In this experiment five cell lines, including MCF7, PC3, HepG2, HT29 and Jurkat were treated with five concentrations of JG-98 (starting from 5 μM with sequential three-fold dilutions) for 24 hours. RNA was isolated and gene expression was assessed using the L1000 platform. For comparison, same cell lines were treated with a bona fide Hsp90 inhibitor, tanespimycin. The effects of JG-98 and tanespimycin on gene expression were compared with effects of other drugs in the Connectivity Map database (for links to the full results of this analysis see Table S1). The similarity of signatures was measured using the connectivity score, a metric that captures the degree of similarity of top up- and downregulated gene sets between two perturbation profiles. As expected, effects of tanespimycin on gene expression were similar to effects of other Hsp90 inhibitors. In fact, top connections, i.e. drugs that show the strongest similarities in the patterns of gene expression changes with such of tanespimycin all were various Hsp90 inhibitors, including geldanamycin, NVP-AUY922, BIIB021 and tanespimycin (Table 1). These inhibitors demonstrated high connectivity scores with tanespimycin (up to 0.95, see links in Table S1). In contrast, Hsp90 inhibitors were not found among drugs that showed reasonable connectivity scores with JG-98 (Table 2). These data clearly indicate that major physiological responses of cells to Hsp90 inhibitors and JG-98 are very different, and therefore compounds of JG-98 series represent an entirely novel pharmacological entity.

**Table 1:** List of top 14 drugs that demonstrate connections with tanespimycin in MCF7 cells.

rank	name	cell line	time	doses	connectivity score
1	geldanamycin	MCF7	24 h	10 µM	0.9349
2	geldanamycin	MCF7	24 h	10 µM	0.9196
3	tanespimycin	MCF7	24 h	0.5 µM	0.916
4	geldanamycin	MCF7	24 h	10 µM	0.9151
5	geldanamycin	MCF7	24 h	3 µM	0.9133
6	geldanamycin	MCF7	24 h	10 µM	0.9116
7	geldanamycin	MCF7	24 h	3 µM	0.9106
8	geldanamycin	MCF7	24 h	10 µM	0.9096
9	geldanamycin	MCF7	24 h	10 µM	0.9083
10	NVP-AUY922	MCF7	24 h	10 µM	0.908
11	geldanamycin	MCF7	24 h	1 µM	0.9043
12	geldanamycin	MCF7	24 h	10 µM	0.9013
13	geldanamycin	MCF7	24 h	10 µM	0.9
14	geldanamycin	MCF7	24 h	1 µM	0.8997

**Table 2:** List of top 14 drugs that demonstrate connections with JG-98 in MCF7 cells.

rank	name	cell line	time	doses		connectivity score
1	SYK-inhibitor	HA1E	24 h	10 µM		0.6493
2	vanoxerine	HA1E	24 h	10 µM		0.6437
3	BRD-K98948170	HA1E	24 h	3.33 µM		0.6374
4	avicin-g	HA1E	24 h	1 µM		0.6353
5	CGP-57380	HA1E	24 h	10 µM		0.6327
6	BRD-K38634661	MCF7	24 h	10 µM		0.6309
7	tigecycline	A375	24 h	3 µM		0.6258
8	KI-8751	HA1E	24 h	10 µM		0.625
9	lonidamine	PC3	6 h	100 µM		0.6231
10	POLR3E	A375	96 h	1 µM		0.6219
11	lavendustin-c	MCF7	6 h	10 µM		0.6219
12	avicin-d	HA1E	24 h	3 µM		0.6204
13	BRD-K26119753	VCAP	24 h	10 µM		0.6183
14	COL-3	HA1E	24 h	10 µM		0.6148

We then compared JG-98 signature to previously generated profiles in the Connectivity Map. Interestingly, the best connectivity score with JG-98 was relatively low, 0.65, reflecting limited similarities in transcription effects of known drugs and bioactive compounds with JG-98. Surprisingly, JG-98 connectivity with other Hsp70 inhibitors not belonging to JG series, including VER-155008 and pifithrin μ, were even lower (0.54 and 0.55, correspondingly, not shown). Nevertheless, effects of JG98 on cancer signaling are specific, since they can be suppressed by pre-incubation with VER-155008 (see below). Therefore, allosteric Hsp70 inhibitors of JG-98 series show unique physiological effects on diverse cancer cells.

To further explore physiological effects of JG-98 in an unbiased way, we treated MCF7 cells with 2 μM of JG-98 for 36 hours, isolated RNA and performed microarray analysis (GEO Series ID GSE93542). Microarray data were analyzed using the Broad Institute software package for gene set enrichment analysis (GSEA) in Boston University Center for Translational Research. GSEA allows deducing pathways up- and downregulated by a treatment based on gene expression data. Analysis of lists of pathways that were significantly up- and downregulated by JG-98 showed 176 downregulated pathways and 19 upregulated pathways (Tables S2 and S3). Of note, the GSEA software is designed in a way that the same gene could be listed as a component of different pathways. Accordingly, certain pathways have a strong overlap in genes that represent them. Therefore, in order to clarify the data we analyzed functional gene families represented in each pathway identified by GSEA and merged them into a more limited number of pathways up- or downregulated in response to JG-98. Among downregulated functional gene families, we identified those controlling (1) the cell cycle, (2) RNA processing and splicing, (3) proteasome, heat shock proteins and CCT chaperones, (4) Cellular energetics, including respiratory chain, ATP synthetase, Crebs cycle and glycolysis, and (5) sterol biosynthesis. Among upregulated gene families we identified (1) unfolded protein response genes, (2) circadian rhythm, and (3) p53/DNA damage response genes.

This pathway analysis further confirmed that physiological effects of JG-98 are significantly different from those of Hsp90 inhibitors. For example, JG-98 downregulates heat shock response, and upregulates unfolded protein response and DNA damage response. In contrast, Hsp90 inhibitors modulate these pathways in opposite directions.

**Figure 1:**
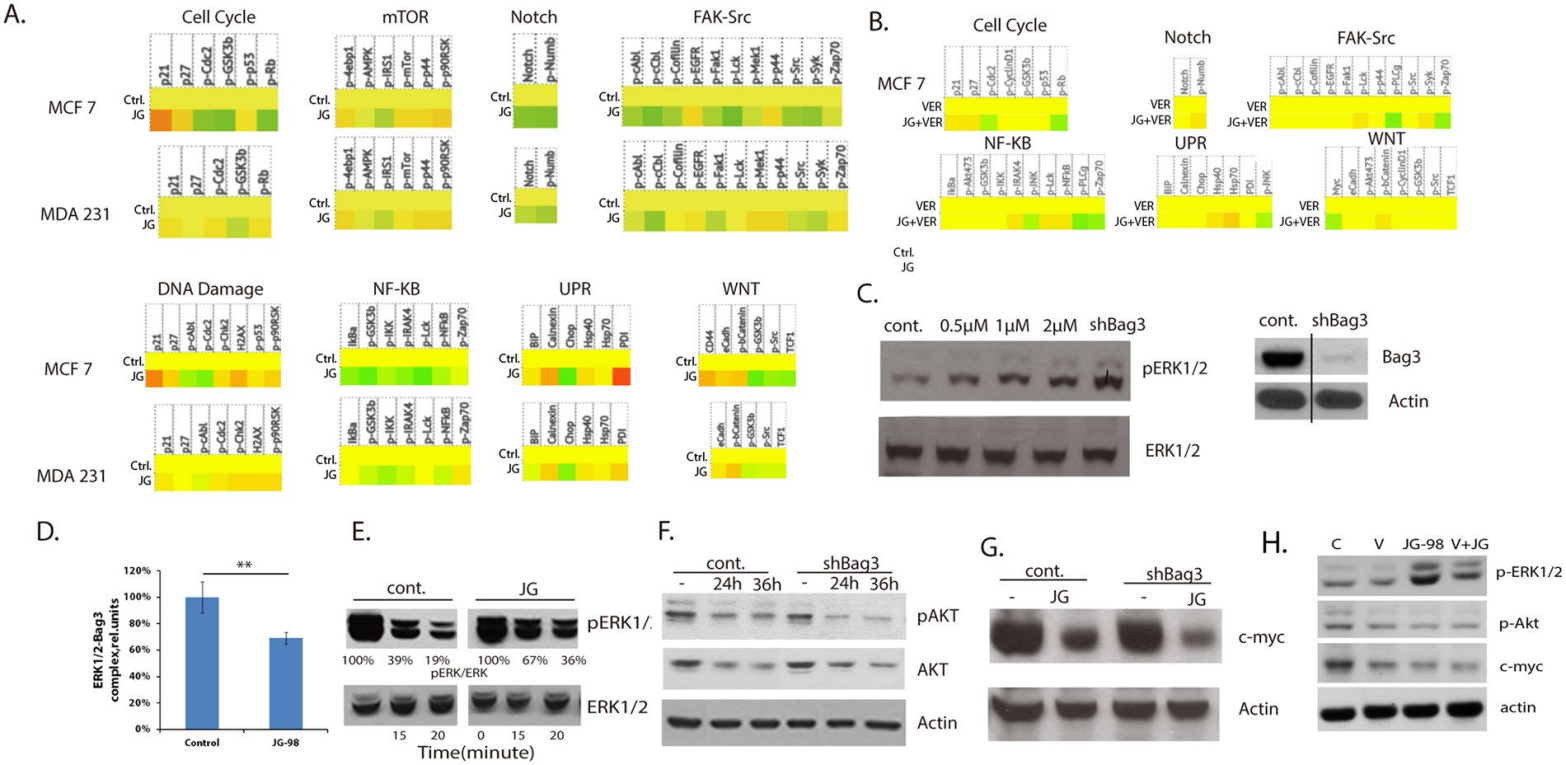
Pathways regulated by JG-98. (**A**) Heat maps demonstrating responses of several signaling pathways to JG-98 in MCF7 and MDA-MB231 cells. Experiment was done using IPAD technology by ActivSignal, Inc. Of note, three gradations of color intensities are presented on the heat map, corresponding to 1.2, 1.8 and 2.4 and higher fold increase or decrease in IPAD values over control. Translation of the IPAD values to actual change in the activity of signaling molecules depends on the target. On average, 1.8 fold change in IPAD values corresponds to 3-fold change in the target activity. (**B**) Preincubation with 10 μM VER-155008 suppresses signaling responses to JG-98. Experiment was done using IPAD technology by ActivSignal, Inc. (**C**) Left panel - JG-98 treatment and Bag3 depletion activates ERK1/2. MCF7 cells were treated for 36 h with indicated concentrations of JG-98, and levels of ph-ERK1/2 and total ERK1/2 were determined in cell lysates by immunoblotting with the corresponding antibody. To deplete Bag3 MCF7 cells were infected with Bag3 shRNA retroivirus, as described in our prior publications^23^. Right panel - shRNA mediated Bag3 depletion. Bag3 levels were determined by immunoblotting cell lysates with Bag3 antibody. This and all other immunoblots were done three times. A typical immunoblot is shown. (**D**) Quantification of effects of JG-98 on association between ERK1/2 and Bag3. Association was studied using the ActivSignal IPAD protein-protein interaction method. Histogram represents an average of three independent experiments. SD values are shown, p < 0.01. (**E**) JG-98 reduces the rate of dephosphorylation of ph-ERK1/2. MCF7 cells were treated with PDB for 7 min to activate ERK1/2 and then further phosphorylation was blocked by incubation of cells with cocktail of inhibitors of respiration (rotenone, 5uM) and glycolysis (2DG 10mM). Cells were collected at indicated time points, and the levels of ph-ERK1/2 and total ERK1/2 were determined as in Fig. 1A. (**F**) JG-98 downregulates AKT in Bag3-independent manner. MCF7 cells were infected with Bag3 shRNA virus or “empty” shRNA vector (see Materials and Methods) prior to treatment with 2 μM of JG-98 for indicated time periods. Levels of Akt and p-Akt were determined in cell lysates by immunoblotting with corresponding antibodies. (**G**) JG-98 downregulates c-myc in Bag3-independent manner. MCF7 cells were infected with Bag3 shRNA virus or “empty” shRNA vector as in Fig. 1G and treated with 2 μM of JG-98 for 36h. Levels of c-myc were determined in cell lysates by immunoblotting with corresponding antibody. (**H**) Preincubation with VER-155008 suppresses effects of JG-98 on ERK, Akt and myc.

To further focus on signaling, we profiled major signaling pathways using the IPAD service provided by ActivSignal, Inc. This service provides information about phosphorylation states, protein levels or cleavage of more than 60 signaling factors that cover more than 20 major signaling pathways. A significant advantage of the ActivSignal methodology is that it is highly sensitive and requires less than 50,000 cells fixed on a well of 96 well plate. MCF7 and MDA-MB231 cells were treated with 2 μM JG-98 for 24 hours, or left untreated, fixed, and sent to ActivSignal, Inc. for signaling pathways profiling. Results of this experiment are presented in Figs 1A and S4. In part, they confirmed results of pathway analysis based on mRNA profiling in MCF7 cells, described above. For example, we observed suppression of the cell cycle progression (upregulation of p21 and p27, and reduction of Rb phosphorylation), activation of the DNA damage response (increased phosphorylation of histone H2AX and Chk2) and activation of UPR (increased levels of calnexin and PDI). Predictably, in MDA-MB231 cells that lack normal p53 response, we observed neither significant upregulation of p21 or p27, nor reduction of phosphorylation of Rb. At the same time, the DNA damage response and UPR were upregulated in MDA-MB231 cells in response to JG-98.

In addition to predicted pathways, this approach identified several signaling pathways that were not identified based on the mRNA profiling. These pathways included (1) suppression of NF-kB, FAK-Src, Notch and WNT, and (2) activation of mTOR and MAPK pathways (Fig. 1A and S). Interestingly, with most pathways (except, predictably, the cell cycle), effects of JG-98 were generally similar in the two tested breast cancer cell lines.

To test whether effects of JG-98 on signaling pathways are related to its inhibitory effects on Hsp70, we preincubated cells with a distinct inhibitor of multiple Hsp70 family members VER-155008, and tested effects of JG-98 in the presence of this inhibitor. Indeed, preincubation with VER-155008 with few exceptions reduced effects of JG-98 on multiple signaling targets in multiple pathways, indicating high specificity of the latter compound (Fig. 1B).

This analysis showed that effects of JG-98 on signaling pathways are both cancer-suppressing (e.g. suppression of NF-kB) and cancer-promoting (e.g. activation of mTOR). Nevertheless, based on extensive prior *in vitro* and *in vivo* data, we know that the overall balance of effects of JG-98 is towards cancer suppression. Importantly, the pathway analysis based on the ActivSignal data helped us to identify the pathway for investigation of Bag3-dependence of the JG-98 effects.

### JG-98 affects cell signaling in Bag3-dependent as well as Bag3-independent manner

JG-98 targets most members of the Hsp70 family, some of which do not interact with Bag3 or other Bag family members, e.g. ER resident Hsp70 BiP or mitochondrial Hsp70. On the other hand, our previous studies suggested that the Hsp70-Bag3 complex is a physiologically significant target for the anti-cancer activity of JG-98^23,24^. To address the role of Hsp70-Bag3 interaction in cellular response to JG-98, we tested whether Bag3 mediates effects of JG-98 on signaling pathways. ActiveSignal data indicated that JG-98 can activate MAP kinase ERK1/2 kinases (Fig. S4). These data were confirmed by immunoblotting of JG-98-treated lysates with anti-p-ERK1/2 antibody (Fig. 1C). Importantly, preincubation with VER-155008 sigificantly suppressed activation of ERK by JG-98 (Fig. 1H), indicating that the latter affects ERKs via inhibition of Hsp70. Originally, we demonstrated that Hsp70 is involved in activation of ERK1/2 through regulation of its de-phosphorylation^31^. Furthermore, it was reported that Bag3 can also directly bind ERK1/2-specific phosphotase and modulate ERK1/2 dephosphorylation^32^. In line with this report, we found that shRNA-mediated Bag3 depletion led to activation of Erk1/2 in MCF7 cells (Fig. 1C). Considering these new and published data together, we suggested that effects of JG-98 on ERK1/2 involve inhibition of the Hsp70-Bag3 module.

It is plausible that JG-98, via binding to Hsp70, can affect interaction between Bag3 and ERK1/2 leading to slower dephosphorylation of the latter. Accordingly, we directly tested if JG-98 affects association of Bag3 with ERK1/2. His-tagged Bag3 was expressed in cells, and cells were incubated with or without 2 μM of JG-98. Standard co-IP experiments were not sensitive enough to study association of His-tagged Bag3 with ERK1/2; therefore, we utilized a significantly more sensitive method to assess interactions between endogenous proteins developed by ActivSignal, Inc. In this assay, MCF7 cells were plated on a 96-well plate and treated with 2 μM of JG-98 for four hours. Treated and control cell were fixed on a plate and incubated with a pair of primary antibodies against ERK1/2 (mouse antibody) and Bag3 (rabbit antibody). Next, cells were incubated with secondary antibodies covalently linked to special oligonucleotides, according to ActivSignal protocol (IPAD protein-protein interaction). In this method protein-protein interaction is detected when primary antibodies associate with interacting molecules and juxtapose secondary antibodies, allowing ligation of oligonucleotides attached to secondary antibodies with a linker oligonucleotide; the ligated product is then detected by Q-PCR.

This approach is several orders of magnitude more sensitive than the pull down assay. Indeed, the ActivSignal approach allowed easy quantitative detection of the complex using 40,000 cells. As negative controls, in these experiments we omitted primary antibodies against either ERK or Bag3. The results were normalized by the amounts of Bag3. This experiment indicated that ERK readily forms complexes with Bag3, and that incubation with JG-98 reduces this association (Fig. 1D).

Next, we tested if JG-98 inhibits dephosphorylation of ERK1/2. Cells were incubated with 2 μM of JG-98, and the rate of ERK1/2 dephosphorylation was measured by method that we have developed previously^31,33^. Briefly, ERK1/2 phosphorylation was activated by incubating cells with Phorbol 12,13-dibutyrate (PDB). To stop further ERK1/2 phosphorylation by the upstream kinases cells were treated with a cocktail of inhibitors of respiration (rotenone) and glycolysis (2DG) (see Materials and Methods). Previously we have demonstrated that this scheme efficiently deplete ATP by 90% within 5 min, which in turn blocks phosphorylation of ERK1/2 by the upstream kinases. Cells were collected at indicated time points following addition of the cocktail of respiration/glycolysis inhibitors, and the levels of remaining phosphorylated ERK1/2 were measured by immunoblotting with the anti-ph-ERK antibody^31^. Indeed, incubation with JG-98 significantly reduced the rate of ERK1/2 dephosphorylation (Fig. 1E), similar to previously reported effect of Bag3 depletion on ERK1/2 dephosphorylation. Therefore, by binding to Hsp70, JG-98 causes dissociation of Hsp70 from Bag3, reduces ERK1/2-Bag3 interaction which in turn suppresses ERK1/2 dephosphorylation, thus increasing ERK1/2 activity.

As noted above, not all effects of JG-98 on signaling pathways may depend on Hsp70-Bag3 interaction. In fact, effects of JG-98 on the Akt pathway, being dependent on inhibition of Hsp70 family members, were Bag3 independent. Previously, it was demonstrated that JG-98 treatment reduces Akt levels^34^. We confirmed it and showed that effects were even stronger when active phosphorylated form of Akt was assessed by immunoblotting with the corresponding antibody (Fig. 1F). Similar downregulation was seen upon incubation with VER155-008. Importantly, JG-98 did not further reduced p-Akt compared to VER155-008 alone (Fig. 1H), strongly suggesting that JG-98 exerts its effect on Akt via Hsp70 inhibition. To check if JG-98-mediated suppression of Akt pathway was dependent on Bag3 we have depleted Bag3 using retrovirus expressing Bag3 shRNA or “empty” shRNA vector (see Materials and Methods) prior to treatment with JG-98. As seen in Fig. 1F, depletion of Bag3 did not reduce JG-98-mediated suppression of either Akt or p-Akt. Moreover, surprisingly, Bag3 depletion even enhanced downregulation of both Akt and p-Akt. This unexpected finding led us to suggest that Bag3 competes with another factor responsible for the JG-98 effect on Akt, and accordingly removal of Bag3 promotes the effect. Obvious candidates were two other major Bag-family members Bag1 or Bag6, both of which were previously shown to promote degradation of various signaling proteins^35,36^. However, depletion of either Bag1 or Bag6 proteins did not reduce dowregulation of Akt by JG-98 (Fig. S1).

Another factor that was dramatically downregulated by JG-98 in Bag3 - independent manner was c-myc (Fig. 1G). This finding was in line with previous report that Hsp70 knockout downregulates c-myc in colon cancer models^11^. C-myc downregulation following incubation with 2 μM of JG-98 was not affected by depletion of Bag3, or silencing of either Bag1 or Bag6 (Fig. S2). Importantly, similar effect on c-myc downregulation was seen with VER-155008 (Fig. 1H), and combination of JG-98 and VER155008 did not further reduce the c-myc level (Fig. 1H). Thus, effects of JG-98 on signaling pathways may involve some other Hsp70 co-chaperone or could be mediated by a distinct Hsp70 family member that does not interact with Bag proteins. We concluded that JG-98 modulates a variety of pathways both in Bag3-dependent and Bag3-independent fashion, and balance of these pathways defines anticancer effects of JG-98.

### Genetic analysis of pathways involved in JG-98-mediated killing of cancer cells

So far we identified a set of pathways that are up- or downregulated in response to JG-98. Further, we focus on identifying among them pathways that affect sensitivity of cancer cells to this drug. Accordingly, we performed pooled shRNA screens in MCF7 and MDA-MB231 cells using shRNA library that targets genes encoding components of signaling pathways. This library covers about 20% of human ORFs. Due to the *in vitro* nature of the screen, we could not investigate effects of JG-98 on metastasis, angiogenesis and other processes that require *in vivo* systems. Nevertheless, our screen provides an important information regarding pathways involved in growth and viability of cancer cells.

MCF7 cells were infected with the lentiviral library at low MOI. Two days post-infection puromycin was added to select cells expressing shRNAs. Cells were then incubated with 3 μM JG-98 for 48 hours. Under these conditions, about 75% of cells died. As control, we used untreated cells. Control cells and cells that survived JG-98 treatment were collected for genomic DNA isolation. Pooled shRNA barcodes were amplified from the genomic DNA mix, sequenced by Ion Torrent, and frequencies of shRNA species presented in treated survivor cells versus un-treated control cells were established (see Materials and Methods; Table S4). To analyze differential occurrence of shRNAs in two cell groups we first established a reference point by using “neutral” shRNAs targeting luciferase present in the library. Occurrence of majority of other shRNAs in two cell groups was similar to luciferase and thus represented “neutral” genes not involved in JG-98 killing. shRNA whose frequencies significantly differed in treated and control samples above the established threshold were further analyzed.

**Figure 2:**
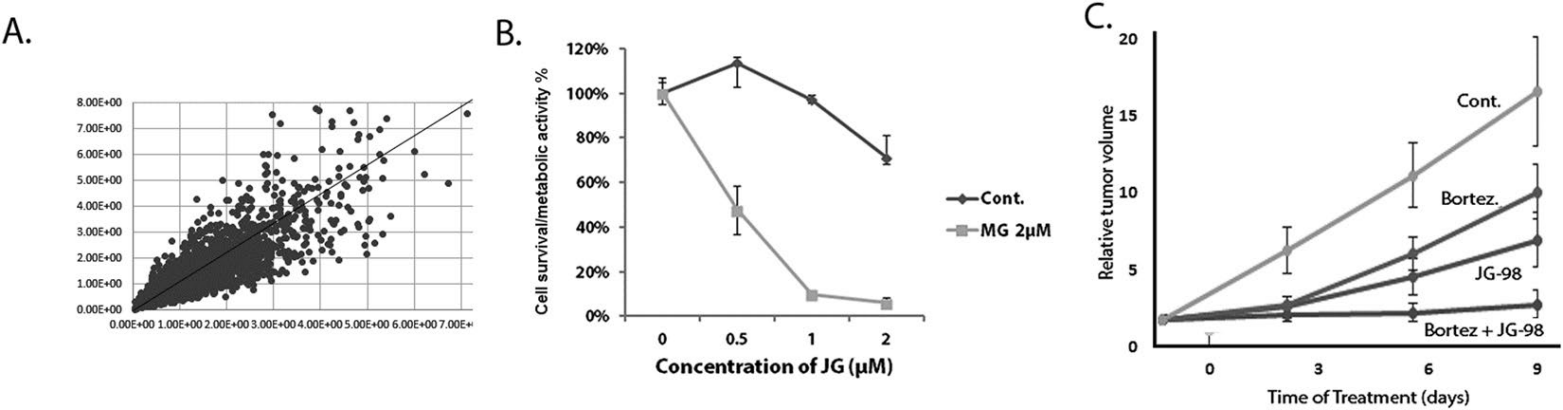
shRNA screening and effects of combinations of JG-98 with proteasome inhibitors. (**A**) Representation of individual shRNA species in JG-98 treated and control samples in two independent experiments. (**B**) Effects of combination of MG132 with JG-98 in MDA-MB231 cells. Sub-toxic concentration of MG132 was determined and effects of JG-98 either alone or in combination with this MG132 concentration were determined by MTT assay. Incubation conditions are described in the text. SD values are shown. (**C**) Effects of combination of bortezomib and JG-98 in the xenograft model of tumor derived from MDA-MB231 cells. In this experiment we used 8 animals in each control and bortezomib-treated groups, and 13 animals in each JG-98 and JG-98 + bortezomib-treated groups. Tumor volumes were calculated based on the tumor size measurements, and increase of tumor volume over time was calculated relative to original volume of each tumor before the start of drug treatment. SD values are shown.

Before comparison of representation of shRNA species in control and treated samples, the software normalizes representation of each shRNA to the size of the sample, i.e. calculates fraction of reads belonging to a specific type of shRNA in the total population of shRNAs in each sample. This normalization allowed us to run correlation analysis of shRNA representation in biological repeats. We compared ratios of each shRNA species in JG-98 treated and control samples in two independent experiments. Figure 2A shows a strong correlation between these ratios (values for ratios for individual shRNAs in two experiments are located along the diagonal line). These data indicate good reproducibility in biological repeats.

Further, we separated shRNAs species into those over-represented in survivor cells, i.e “protective” shRNAs that by silencing corresponding genes provide protection of cells from JG-98 killing and those under-represented in survivor cells “sensitizing shRNAs”, i.e shRNAs that by silencing corresponding genes sensitize cells to killing by JG-98.

At the next step, identified genes that significantly affect sensitivity to JG-98 were sorted according to biological pathways using Ingenuity pathway analysis software (Table 3). The most under-represented shRNAs species in survivor cells were those targeting components of the proteasome pathway. In fact, among about 250 hits we found twelve genes that encode subunits of both 20S and 19S components of the 26S proteasome. Other highly represented pathway was RNApol II, where targeting of six genes encoding its components significantly sensitized cells to JG-98. Silencing of several other pathways also provided sensitization to JG-98 killing. Table 3 lists components of these pathways identified in MCF7 and MDA-MB231 cells, including translation, Wnt, TNF-NF-kB, Golgi transport, cullin E3 ubiquitin ligase, and TGF-β. Components of proteasome, RNApol II and translation initiation were highly represented in both cell lines, while other pathways were under-represented in MDA-MB231 cells (Table 3). Importantly, NF-kB and WNT pathways that significantly protect from JG-98 according to the genetic data (Table 3) were suppressed upon incubation with JG-98 (see Fig. 1A). In other words, suppression of these pathways by JG-98 facilitates the responses to this drug, thus establishing positive feedback loops.

**Table 3:** Genes that affect sensitivity to JG-98 sorted according to major pathways.

Major Pathway	Protector shRNA	Sensitizing shRNA
Proteasome		PSMA1 PSMA2 PSMA3 PSMA6 PSMA7 PSMB2 PSMB4 PSMB6 PSMC1 PSMC2 PSMC3^*^ PSMC6^*^ PSMD1 PSMD11^**^ PSMD12^**^ PSMD2^**^ PSMD3 PSMD6^**^ PSMD8^**^ PSMF1^**^
RNApol		POLR2A POLR2B POLR2D POLR2F POLR2I^*^
TGF	FNTA^*^ SMAD2^*^ TGFBR1^*^	USP9X STRAP
WNT	GSK3A^*^	FZD3* FZD4* FZD7* FZD8^*^ FGF8^*^ TCF7L1^*^
Golgi	ARFGEF2^*^	ARF1^*^ ARFGAP3^*^
Cullin	c-myc^*^	SKP1^**^ RBX1^*^ Fbxw7^*^ P21^*^ P27^*^
Translation initiation		EIF1AX EIF1AY^*^ EIF2B5 EIF2S2^*^ EIF3A EIF4BP1^**^

No * - Genes identified in both MCF7 and MDA-MB231 cells.

^*^Genes identified only in MCF7 cells.

^**^Genes identified only in MDA-MB231 cells.

### Identifying drugs that synergize with JG-98

Many new potentially promising anti-cancer compounds suffer from insufficient potency, poor therapeutic index or development of resistance. Some of these problems could be alleviated by combining the compounds with known anti-cancer drugs. Indeed, monotherapy rarely provides sufficient efficiency and instead drug cocktails are universally used in clinic. There have been multiple attempts to develop rational approaches towards design of synergistic combinations to enhance the potency. Establishing these combinations can define the scope of preclinical studies and inform the design of future clinical trials. Here we approach this problem by analyzing pathways involved in JG-98-mediated killing of cancer cells.

We decided to focus on the proteasome pathway that was highly represented in both MCF7 and MDA-MB231 cells, and to validate the prediction that inhibition of proteasome should sensitize cancer cells to JG-98. Our validation efforts were focused on MDA-MB231 cells that represent triple-negative type of breast cancer (TNBC). The rational for this choice was that TNBC are especially sensitive to proteasome inhibition^37^. Similarly, we found that TNBC lines are hypersensitive of JG-98 (not shown). Therefore, we expected to achieve an additional level of cancer specificity by treating TNBC cells with combination of a proteasome inhibitor and JG-98.

We identified a maximal sub-toxic concentration of the proteasome inhibitor MG132, and cells were treated for 48 hours with combinations of various concentrations of JG-98 with the sub-toxic concentration of MG132. Cell viability/metabolic activity was measured by the MTT assay in 96-well format. Figure 2B shows that while MG132 alone did not affect cells viability, it significantly potentiated effects of JG-98 on cell viability. These data clearly indicate that proteasome inhibition synergistically enhances the JG-98-mediated killing of the MDA-MB231 cells. Of note, similar results were obtained with another breast cancer cell line, BT474 (Fig. S3).

We next validated this finding in *in vivo* experiments using a xenograft cancer model. Of note, effects of JG-98 on growth in various xenograft tumor models and its specificity towards Hsp70-mediated signaling pathways have been reported in our previous publications^23–25^. For these tests, we used bortezomib instead of MG132, since it is an FDA-approved drug. 1.5 × 10^6^ MDA-MB231 cells were injected into nude mice subcutaneously, and when tumors reached 150mm^3^, animals were treated with 1mg/kg bortezomib, 5mg/kg JG-98 or combination of bortezomib and JG-98. Vehicle-treated animals were used as control. Bortezomib and JG-98 caused suppression of tumor growth even when injected alone. Importantly, their combination caused a significantly stronger suppression of tumor growth (Fig. 2C), demonstrating that this combination is more potent than the individual drugs alone in an *in vivo* cancer model. Importantly, this result provides a strong validation for the approach of using *in vitro* shRNA screens for rational design of potent drug combinations.

**Figure 3:**
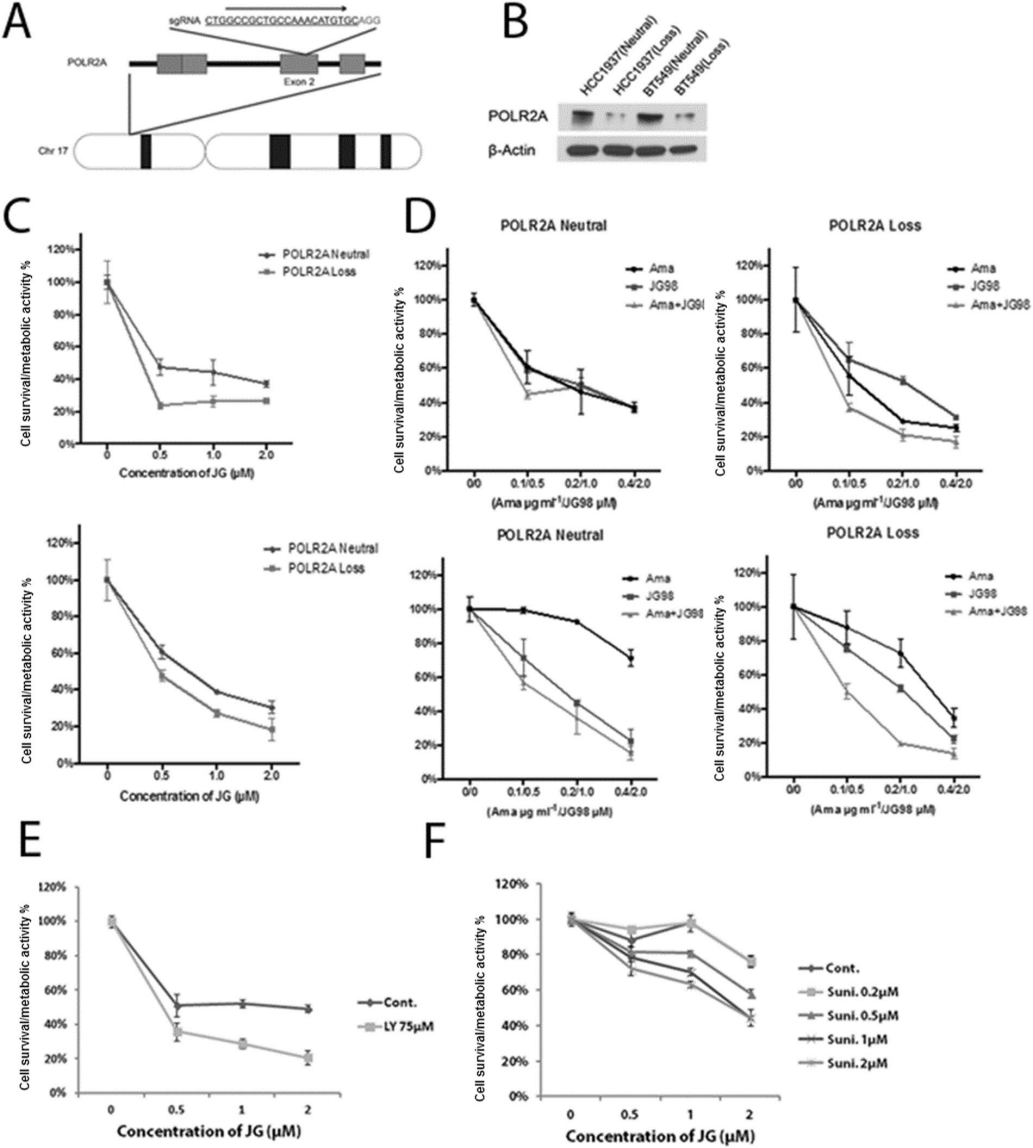
Combination of JG-98 with RNApol inhibitors. (**A**) Schematic illustration of the Cas9/sgRNA-targeting site in the POLR2A gene. The protospacer-adjacent motif (PAM) sequence is highlighted in red. (**B**) Protein levels of POLR2A in parental and isogenic POLR2A-loss HCC1937 and BT549 cell lines. β-actin is used as a control. (**C**) The sensitivity of the POLR2A-neutral and POLR2A-loss cell lines to the treatment of JG-98 in the presence of the sub-toxic concentration of α-amanitin. 0.1 µg/ml and 0.2 µg/ml of α-amanitin were used to treat HCC1937 and BT549 cell lines, respectively. (**D**) Synergistic effect of combined treatments of JG-98 and α-amanitin in human TNBC cells with hemizygous loss of POLR2A. Parental and POLR2A-loss TNBC cells were treated with JG-98 and α-amanitin at indicated concentrations. Effects of combination of α-amanitin with JG-98 in normal and POLR2A haploinsufficient cell line HCC1937. (**E**) Effects of combination of LY294002 with JG-98 in MDA-MB231 cells. (**F**) Effects of combination of sunitinib with JG-98 in MDA-MB231 cells. SD values are shown in all panels of Fig. 3.

A distinct opportunity for a potent drug combination that came out from the genetic screen was combination of JG-98 with RNApolII inhibitors. In fact, results of the genetic screen showed that depletions of multiple RNApolII genes, including POLR2A, sensitized to JG-98 both MCF7 and MDA-MB231 cells. Recently it was reported that in cancers with deletion of p53, a gene encoding RNA polymerase subunit POLR2A is often co-deleted^38^. Such cancers become hypersensitive to an inhibitor of RNA polymerase α-amanitin, which represent a paradigm for specific targeting of cancers with p53 deletions^39^. We hypothesized that inhibition of RNA polymerase could make cells more vulnerable to Hsp70 inhibition, and accordingly α-amanitin may synergize with JG-98. MDA-MB231 cells were very resistant to α-amanitin for unknown reason, therefore, to test our hypothesis we used two other cell lines derived from triple negative breast cancer, including HCC1937 and BT549. Each line has two copies of p53 and POLR2A genes. To specifically address the role of POLR2A in the drug sensitivity, the isogenic cell lines harboring hemizygous deletion of POLR2A (POLR2A-loss lines) were generated from the parental cell lines HCC1937 and BT549 (POLR2A-neutral lines) (Fig. 3A). Mono-allelic deletion of POLR2A resulted in significant reduction of its protein expression levels in both of the POLR2A-loss cell lines in comparison with their parental POLR2A-neutral lines (Fig. 3B). To validate the predicted combination, we treated the isogenic pairs of HCC1937 and BT549 cells with combinations of α-amanitin and JG-98 at various concentrations for 48 hours, and measured cell survival/metabolic activity by the MTT assay. When treated with sub-toxic concentrations of α-amanitin (0.1-0.2 µg/ml), both of the POLR2A-loss cell lines exhibited significantly higher sensitivity to the treatment with JG-98 (Fig. 3C). Combined treatment of JG-98 and α-amanitin at varying concentrations shows that indeed a-amanitin demonstrates synergy with JG-98, and the effect was especially pronounced in cells with POLR2A haploinsufficiency (Fig. 3D). This experiment suggests that Hsp70 inhibitors of JG-98 series could be effectively used in combination with inhibitors of RNApol II for specific treatment of cancers with p53 deletion.

Rational design of drug combination utilizing genetic screen information described above has a bias as it is based on our understanding of pathways. Here, we developed a complementary approach to search for drug combinations based on gene expression analysis. As noted above, the Connectivity Map project from Broad Institute uses gene expression analysis to establish patterns of genes that are up- or downregulated in response to all known drugs in multiple cancer cell lines. We combined the power of Cmap analysis with information obtained in our genetic screen to search for drugs that may significantly increase the potency of JG-98 action. Accordingly, we plugged limited gene sets (genes that affect major pathways) from our shRNA screen analysis to the Cmap software and obtained a list of drugs that demonstrate connectivity. We focused on 40 drugs that showed top connectivity scores. To streamline the analysis, among these drugs we defined groups of functionally similar drugs. Two groups were identified, including nine inhibitors of PI3K-Akt pathway, and five inhibitors of multiple tyrosine kinase receptors, RTK (Tables 4 and S5). Next, as examples, we have chosen an Akt inhibitor LY294002 and an RTK inhibitor sunitinib to investigate whether these drugs can potentiate cell killing by JG-98. Accordingly, MDA-MB231 cells were plated in 96-well plate, incubated with these inhibitors at sub-toxic concentrations in combination with increasing concentrations of JG-98, or with similar concentrations of JG-98 alone, and cell viability was measured by the MTT assay. Indeed, both LY294002 and sunitinib demonstrated significant synergy with JG-98 in cell culture (Fig. 3E,F). Of note, similar results were obtained with another breast cancer cell line, BT474 (Fig. S3). These data indicate that combination of unbiased gene expression analysis with pooled shRNA screens provide a powerful tool for a rational approach to drug combinations.

**Table 4:** Selected drugs among top 40 connections predicted to sensitize cells to JG-98 according to analysis of the Connectivity Map.

rank	cmap_id	cmap_name	score	Target
1	BRD-K13646352	midostaurin	99.191	TKR
8	BRD-K60623809	SU-11652	97.695	TKR
10	BRD-K12184916	NVP-BEZ235	97.412	PI3K
12	BRD-K67868012	PI-103	97.29	PI3K
13	BRD-K06750613	GSK-1059615	97.06	PI3K
14	BRD-K67075780	TGX-115	96.911	PI3K
17	BRD-K68065987	MK-2206	96.68	Akt
26	BRD-K97365803	PI-828	95.681	PI3K
29	BRD-K63068307	ZSTK-474	95.232	PI3K
30	BRD-K67566344	KU-0063794	95.048	Mtor
35	BRD-K69932463	AZD-8055	94.754	mTOR
36	BRD-K40255344	tyrphostin-A9	94.743	TKR
37	BRD-K59469039	AG-879	94.684	TKR
39	BRD-M64432851	sunitinib	94.448	TKR
40	BRD-A11678676	wortmannin	94.352	mTOR

Inhibitors of RTKs and PI3K-Akt-mTOR are indicated.

## Discussion

Here we used combinations of various versions of gene expression analysis and genetic screens to understand physiological effects of the novel class of Hsp70 inhibitors exemplified by JG-98. Our first goal was to compare physiological effects of JG-98 with those of Hsp90 inhibitors using gene expression analysis based on the Connectivity map platform developed by Broad institute. This approach demonstrated that there is little similarity between effects of JG-98 and Hsp90 inhibitors. Furthermore, effects of JG-98 are also significantly different from effects of any existing drugs pointing towards its uniqueness. Accordingly, this series of compounds may have improved anti-cancer activity when compared to Hsp90 inhibitors.

JG-98-like inhibitors interact with a recently described allosteric site on the ATPase domain of Hsp70 and freeze the latter in the ADP-bound form, which in turn leads to dissociation of Bag-family cofactors, including Bag3^24^. Here, we first identified pathways affected by JG-98, and then assessed the role of disruption of Hsp70-Bag3 interaction in pathway regulation by this compound. As exemplary pathway, we focused on the ERK1/2 activation, which previously was reported to be regulated by Bag3. ERK1/2 can directly interact with Bag3, which promotes dephosphorylation and inactivation of the former by the MKP3 phosphatase^32^. To quantitatively assess Bag3-ERK1/2 interaction we utilized a novel method offered as kit by ActivSignal, since conventional co-IP was not sufficiently sensitive. The ActivSignal kit was at least 1000 times more sensitive and provided reproducible quantification of the ERK1/2-Bag3 interaction. Here, we demonstrate that JG-98 causes dissociation of ERK1/2 from Bag3 and slows down the dephosphorylation. In a related study, we found that interaction of other kinases, e.g. Lats1, JNK or p38 with Bag3 is modulated by interaction with Hsp70, since it was significantly reduced by deletion of the Hsp70-interacting domain (in preparation). Accordingly, effects of JG-98 on interaction of Bag3 with kinases could be a general mechanism of its action.

Effects of JG-98 on pathways were not limited to disruption of Hsp70-Bag3 interaction. An example of such effect was JG-98-caused downregulation of Akt. Depletion of Bag3 neither mimicked nor reversed downregulation of Akt following incubation with JG-98. Similar effect was seen with downregulation of c-myc. Probably in these cases JG-98 acts by enhancing association of Hsp70 with substrate polypeptides and recruitment of the ubiquitin ligase CHIP, which in turn promotes ubiquitination and degradation of Hsp70-bound substrates^40,41^. Interestingly, effects of JG-98 on c-myc downregulation were very strong. Though here we focused on breast cancer models, these effects on c-myc suggest that JG-98 series of compounds could be potent against cancers that show c-myc upregulation, like lung cancers. Overall this study demonstrate that JG-98 series of compounds have multiple effects on cancer-related signaling pathways via both Bag3-dependent and –independent mechanisms.

Here, we also developed approaches to design potent combinations of known drugs with JG-98. Via shRNA screens, we established gene patterns that modulate cell sensitivity to JG-98 and used this information to design drug combinations. First approach involved a straightforward pathway analysis. Using this approach we identified that downregulation of the proteasome pathway sensitizes to JG-98, and predicts that proteasome inhibitors should synergize with this compound. This prediction was validated using both *in vitro* and *in vivo* models. Notably, TNBC was shown to be highly sensitive to proteasome inhibitors. Here, we found that the TNBC line MDA-MB231 is also very sensitive to JG-98 both *in vitro* and xenograft model. Accordingly, the combination of JG-98 series with proteasome inhibitors could be a potent combination for treatment of this extremely aggressive cancer type.

We also tested effects of combination of JG-98 with inhibitors of RNApol II. The latter inhibitors were demonstrated to be especially effective against cancers with p53 deletion, which very often is co-deleted with POLR2A gene^39^, which makes these cells vulnerable to RNApol II inhibitors like α-amanitin. Indeed, we found that a-amanitin synergizes with JG-98 in killing TNBC lines, and this effect was more pronounced in clones with POLR2A haploinsufficiency. α-amanitin is a very toxic compound, but its antibody-based delivery significantly reduces toxic effects and is effective in animal cancer models^38^. Accordingly, upon development, these RNApol II inhibitors could be effectively combined with JG-98 series of Hsp70 inhibitors. Overall, these experiments indicate this approach to design drug combinations is effective.

Another approach that we introduce here involves a combination of genetic screen data and gene expression data. At the first step, we identified gene sets that either sensitize to JG-98, or protect from this compound. Then we asked a question whether there are known drugs that enhance expression of the sensitizing genes and reduce expression of the protective genes. This approach is less biased than the pathway analysis approach, since it is not dependent on our definition of the pathways. Here we used a Connectivity Map database which has gene expression data in response to all FDA-approved drugs in multiple cancer cell lines. We plugged gene sets identified in the genetic screen in the Connectivity Map database, which predicted that inhibitors of the PI3K-Akt pathway and inhibitors of RTKs should sensitise to JG-98. In fact we validated these predictions with LY294002 and sunitinib. These findings will narrow the focus of further preclinical and clinical studies of JG-98 series of Hsp70 inhibitors. Furthermore, these approaches to design drug combinations may be applied to a variety of novel anti-cancer drugs. Overall, a comprehensive analysis of effects of new drug prototypes on cancer cell physiology, similar to analysis performed in this study, could be generally useful to direct pre-clinical and clinical studies.

## Materials and Methods

### Cell cultures, reagents and treatments

MCF7 and MDA-MB231 cells were from American Type Culture Collection. Cells were grown in DMEM supplemented with 10% fetal bovine serum; 100 units mL^−1^ penicillin, and 0.1 mg mL^−1^ streptomycin. LentiX293T (Clontech; Madison. WI) were grown in same media except FBS was heat inactivated for 30 min at 56 °C, and media was supplemented with L-Glutamine (Corning; 2 mM final). All cells were grown in a humidified environment at 37 °C with 5% CO_2_.

### Reagents

JG-98 was a kind gift of Dr. Dr. J.E. Gestwicki (University of California at San Francisco, San Francisco, CA), Phorbol 12,13-dibutyrate, PDB(Sigma. Cat. #P1269, St. Louis, MO.) was made as a 2 mM stock solution in dimethyl sulfoxide and added to cells at a final concentration of 1 μM for 7 min., Rotenon (Sigma R8875; St. Louis, MO) was made as a 10 mM stock solution in dimethyl sulfoxide and added to cells at a final concentration of 5 μM, 2-deoxy-D-glucose, 2DG (Sigma D-3179; St. Louis, MO) was made as a 1 M stock solution in water and added to cells at a final concentration of 10 mM, bortezomib (Selleckhem, Cat.#S-1013; Houston, TX), MG-132 (Biomol, Cat.#PI-102; Plymouth Meeting, PA), SB431542 (Selleckhem, Cat.#S-1067; Houston, TX), XAV-939 (Selleckhem, Cat.#S-1180; Houston, TX), VER-155008 (Sigma, SML0271), sunitinib (Selleckhem, Cat.#S7781; Houston, TX), LY294002 Cayman Chemical, Cat.#70920; Ann Arbor, MI); Lipofectamine RNAiMac (Invitrogene; Cat.#13778–030).

### Immunoblotting

Cells lysates were prepared as described^42^. Antibodies used in the study were from Cell Signaling (Danvers, MA): β-actin (Cat.#3700); Akt (Cat. #9272); p-AKT Ser473 (Cat. #9271); p44/42 ERK Rabbit (Cat. #9102); phospho-p44/42 ERK -Thr202/Tyr204 (Cat. #9101). Antibody against p44/42 ERK mouse (Cat. #14–9108) were from AffimetrixeBioscience, San Diego, CA, antibody against Bag3 (Cat.#ABC277 were from Millipore,Danvers MA.

Quantification of blots was performed using Quantity One software (NIH ImageJ).

### RNA isolation

RNA was isolated using Qiagen RNeasy Mini kit (Cat. #74104) according to manufacture recommendations.

### Genomic DNA isolation

DNA was isolated using Qiagen DNeasy Blood&Tissue kit (Cat. #69504) according to manufacture recommendations.

### RNA microarray

Microarray data were analyzed using the Broad Institute software package for gene set enrichment analysis (GSEA) in Boston University Center for Translational Research.

### MTT assay

Cell survival/metabolic activity was determined by CellTiter 96 Aqueous One Solution Assay (Promega; Madison, WI Cat. #G3580) according to the manufacturer’s instructions. Cells were seeded in 96-well plates in triplicates (6 × 10^3^ per well) and incubated with different concentrations of drugs for 48 hours or left untreated. Plates were read using BioTek Synergy HT Multi-Mode Microplate Reader. All measurments were done in triplicates.

### Retrovirus and lentivirus production

Retroviruses expressing shRNAs, empty vector, or control enhanced green fluorescent protein (EGFP) were produced by co-transfection of HEK293T cells via Lipofectamine 2000 with plasmids expressing retroviral genomes, packaging proteins Gag-Pol, and the VSV-G protein. Lentiviral vectors were produced by co-transfection of HEK293T cells with plasmids expressing lentiviral packaging plasmid, psPAX2, VSV-G expression plasmid, pMD2.G, and either control EGFP or alternative transgene-expressing constructs. At 48 h after transfection, retrovirus or lentivirus - containing supernatants were collected and frozen at −80 °C. Cells plated in 35 mm dishes were infected with 100 ul of retrovirus, 900 ul media plus 10 μg/mL hexadimethrine bromide (Sigma; Cat. #H-9268) overnight, washed, and selected with puromycin (1 μg/mL) 48 h after infection. Retroviral vectors expressing EGFP were used as an infection efficiency indicator: usually about 75% of cells were fluorescent 2 d after infection. Bag3 retrovirus was described in ref.^23^.

All RNAi were from Dhamacon (Lafayette, CO). RNAi against Bag1 (Cat. #D-003871-01) and against Bag6 (Cat. #D-005062-01) were used according to manufacture protocol.

### Pooled shRNA screens using shRNA

shRNA library was from Cellecta, Inc. (Mountain View, CA). Specifically we have used Module 1 Decipher shRNA library (Cat. #DHPAC-M1-P) Experiment was done according to manufacture recommendations. Briefly, lentivirus expressing shRNA library was produced as described above. We used three 15cm dishes at 80% confluency to transfect 30ug of shRNA library. Supernatant containing lentivirus was used to infect cells; we titrated virus to obtain 50% infectivity as judged by observation of cells under florescence microscope to detect RFP-positive (infected) cells. 48 hours post-infection puromycin was added to select cells expressing lentivirus for additional 48 hours upon which cells were splitted into two groups: control cells were left untreated and experimental group was incubated with JG-98 for additional 3 days which led to 75% of cell death. Control cells and cells that survived JG-98 treatment were collected and procecced for genomic DNA isolation and subsequent bar cod amplification according to manufacture recommendation.

### ERK de-phosphorylation assay

De-phosphorylation assay was performed as described previously^31,33^. Briefly, cells were treated with PDB for 7 min to activate ERK1/2 and then further phosphorylation was blocked by incubating cells in the presence of inhibitor of respiration (rotenone, 5uM) and glycolysis (2DG 10mM). This treatment causes ATP depletion by 90% within 3 min, which in turn blocks phosphorylation of ERK1/2 by the upstream kinases. Cells were collected in 5 min intervals following the ATP depletion, and the levels of remaining phosphorylation of ERK1/2 were measured by immunoblotting with the anti-p-ERK antibody

### IPAD protein-protein interaction assay

The IPAD protein-protein interaction assay was done according to the protocol and using reagents (conjugated secondary antibodies and buffers) provided by ActivSignal, Inc. Briefly, the idea of the method is described in the Results section.

### Xenograft cancer model with MDA-MB231

Animal maintenance and experiments were conducted in compliance with the guidelines of the Institutional Animal Care and Use Committee. Protocol approval by IACUC AN-14751.2016.03. Briefly, cells were trypsinized, mixed at 1:1 ratio with matrigel (BD Scientific, San Jose, CA, USA) and 1.5 million cells were injected subcutaneously into 6-week-old female NCR nude mice (Taconic, Hudson, NY, USA). Tumor growth was monitored twice a week by caliper.

### Connectivity Map

The Broad Institute platform L1000 was used to study limited gene expression patterns in multiple cell lines. This system uses a luminex-based technology to assess expression of 1000 so-called landmark genes, which represent patterns of global gene expression.

Series of five concentrations of JG-98 starting from 5 μM with sequential three-fold dilutions were incubated with five cell lines, including MCF7, PC3, HepG2, HT29 and Jurkat for 24 hours in triplicates, RNA was isolated and results were run over the Broad Institute platform. For comparison, in parallel similar tests with same cell lines and under similar conditions were performed with an Hsp90 inhibitor tanespimycin.

For each perturbation, a gene signature of the top up- and downregulated genes was derived using standard L1000 differential expression processing steps. Gene signatures of JG-98 and tanespinmycin were interrogated for similarities to other signatures in the Connectivity Map perturbation database using the tool *sig_query* in the Broad LINCS CMAP environment, C3.

## Electronic supplementary material

Supplementary Information 

## References

[1] Lindquist S, Craig EA (1988). The heat-shock proteins. Annual Review of Genetics..

[2] Michels AA (1997). Hsp70 and Hsp40 chaperone activities in the cytoplasm and the nucleus of mammalian cells. Journal of Biological Chemistry..

[3] Stege GJ (1994). On the role of hsp72 in heat-induced intranuclear protein aggregation. International Journal of Hyperthermia..

[4] Jaattela M (1999). Escaping cell death: Survival proteins in cancer [Review]. Experimental Cell Research..

[5] Jolly C, Morimoto RI (2000). Role of the heat shock response and molecular chaperones in oncogenesis and cell death. J Natl Cancer Inst..

[6] Mosser DD, Morimoto RI (2004). Molecular chaperones and the stress of oncogenesis. Oncogene..

[7] Calderwood SK, Khaleque MA, Sawyer DB, Ciocca DR (2006). Heat shock proteins in cancer: chaperones of tumorigenesis. Trends Biochem Sci..

[8] Ciocca DR, Calderwood SK (2005). Heat shock proteins in cancer: diagnostic, prognostic, predictive, and treatment implications. Cell Stress Chaperones..

[9] Meng L (2011). Heat shock protein Hsp72 plays an essential role in Her2-induced mammary tumorigenesis. Oncogene..

[10] Gong, J. *et al*. Targeting the hsp70 gene delays mammary tumor initiation and inhibits tumor cell metastasis. *Oncogene* (2015). 10.1038/onc.2015.1 PMC7331470 25659585

[11] Tao Y (2016). Hsp70 exerts oncogenic activity in the Apc mutant Min mouse model. Carcinogenesis..

[12] Hunt CR (2004). Genomic instability and enhanced radiosensitivity in Hsp70.1- and Hsp70.3-deficient mice. Mol Cell Biol..

[13] Wang Z (2011). Induction of heat shock protein 70 inhibits ischemic renal injury. Kidney Int..

[14] McConnell KW (2011). The role of heat shock protein 70 in mediating age-dependent mortality in sepsis. J Immunol..

[15] Rerole AL (2011). Peptides and aptamers targeting HSP70: a novel approach for anticancer chemotherapy. Cancer Res..

[16] Leu JI (2009). A small molecule inhibitor of inducible heat shock protein 70. Mol Cell..

[17] Chang L (2011). Chemical screens against a reconstituted multiprotein complex: myricetin blocks DnaJ regulation of DnaK through an allosteric mechanism. Chem Biol..

[18] Huryn DM (2011). Chemical methodology as a source of small-molecule checkpoint inhibitors and heat shock protein 70 (Hsp70) modulators. Proc Natl Acad Sci USA.

[19] Braunstein MJ (2011). Antimyeloma Effects of the Heat Shock Protein 70 Molecular Chaperone Inhibitor MAL3-101. J Oncol..

[20] Massey AJ (2010). A novel, small molecule inhibitor of Hsc70/Hsp70 potentiates Hsp90 inhibitor induced apoptosis in HCT116 colon carcinoma cells. Cancer Chemother Pharmacol..

[21] Rodina A (2013). Identification of an allosteric pocket on human hsp70 reveals a mode of inhibition of this therapeutically important protein. Chem Biol..

[22] Koren J (2012). Rhodacyanine derivative selectively targets cancer cells and overcomes tamoxifen resistance. PloS one..

[23] Colvin TA (2014). Hsp70-Bag3 interactions regulate cancer-related signaling networks. Cancer Res..

[24] Li X (2015). Validation of the hsp70-bag3 protein-protein interaction as a potential therapeutic target in cancer. Mol Cancer Ther..

[25] Gabai VL (2016). Anticancer Effects of Targeting Hsp70 in Tumor Stromal Cells. Cancer research..

[26] Masarwa M, Donin N, Ziporen L, Fishelson Z (2009). Silencing of the Mitochondrial Hsp70 Mortalin as an Adjuvant Cancer Therapy. Annals of Oncology..

[27] Na Y (2016). Stress Chaperone Mortalin Contributes to Epithelial-to-Mesenchymal Transition and Cancer Metastasis. Cancer Research..

[28] Lee AS (2007). GRP78 induction in cancer: Therapeutic and prognostic implications. Cancer Research..

[29] Li JZ, Lee AS (2006). Stress induction of GRP78/BiP and its role in cancer. Current Molecular Medicine..

[30] Powers MV, Clarke PA, Workman P (2008). Dual targeting of HSC70 and HSP72 inhibits HSP90 function and induces tumor-specific apoptosis. Cancer cell..

[31] Yaglom J, O’Callaghan-Sunol C, Gabai V, Sherman MY (2003). Inactivation of dual-specificity phosphatases is involved in the regulation of extracellular signal-regulated kinases by heat shock and hsp72. Mol Cell Biol..

[32] Falco A (2012). BAG3 controls angiogenesis through regulation of ERK phosphorylation. Oncogene..

[33] Meriin AB (1999). Protein damaging stresses activate JNK via inhibition of its phosphatase: A novel pathway controlled by Hsp72. Mol.Cell. Biol..

[34] Li, X. *et al*. Analogs of the Allosteric Heat Shock Protein 70 (Hsp70) Inhibitor, MKT-077, as Anti-Cancer Agents. *ACS Med Chem Lett*. **4** (2013). 10.1021/ml400204n PMC3845967 24312699

[35] Casson J, McKenna M, High S (2016). On the road to nowhere: cross-talk between post-translational protein targeting and cytosolic quality control. Biochemical Society transactions..

[36] Behl C (2016). Breaking BAG: The Co-Chaperone BAG3 in Health and Disease. Trends in pharmacological sciences..

[37] Petrocca F (2013). A genome-wide siRNA screen identifies proteasome addiction as a vulnerability of basal-like triple-negative breast cancer cells. Cancer cell..

[38] Liu Y (2015). TP53 loss creates therapeutic vulnerability incolorectal cancer. Nature..

[39] Liu Y, Wang L, Lu X (2015). A new way to target p53-defective colorectal cancer. Future oncology (London, England)..

[40] Assimon VA, Gillies AT, Rauch JN, Gestwicki JE (2013). Hsp70 protein complexes as drug targets. Curr Pharm Des..

[41] Wang AM (2013). Activation of Hsp70 reduces neurotoxicity by promoting polyglutamine protein degradation. Nat Chem Biol..

[42] Yaglom JA, Gabai VL, Sherman MY (2007). High levels of heat shock protein Hsp72 in cancer cells suppress default senescence pathways. Cancer Res..

