# Author Correction: Cancer cell responses to Hsp70 inhibitor JG-98: Comparison with Hsp90 inhibitors and finding synergistic drug combinations

**DOI:** 10.1038/s41598-018-24992-x

**Published:** 2018-05-02

**Authors:** Julia A. Yaglom, Yongmei Wang, Amy Li, Zhenghu Li, Stephano Monti, Ilya Alexandrov, Xiongbin Lu, Michael Y. Sherman

**Affiliations:** 1 Boston University School of Medicine, Boston, United States; 2 Center of Diagnosis and Treatment of Breast Disease, The Affiliated Hospital of Qingdao University, Qingdao, China; 3 MD Anderson Cancer Center, Houston, USA; 4 ActivSignal, Watertown, MA USA; 5 Department Molecular Biology Ariel University, Ariel, Israel

Correction to: *Scientific Reports* 10.1038/s41598-017-14900-0, published online 14 February 2018

In the original version of this Article, Michael Y. Sherman was incorrectly affiliated with ‘Boston University School of Medicine, Boston, United States’. The correct affiliations are listed below:

Boston University School of Medicine, Boston, United States

Julia A. Yaglom, Amy Li & Stephano Monti

Center of Diagnosis and Treatment of Breast Disease, The Affiliated Hospital of Qingdao University, Qingdao, China

Yongmei Wang

MD Anderson Cancer Center, Houston, USA

Zhenghu Li & Xiongbin Lu

ActivSignal, Watertown, MA, USA

Ilya Alexandrov

Department Molecular Biology Ariel University, Ariel, Israel

Michael Y. Sherman

This error has now been corrected in the PDF and HTML versions of the Article.

