# Supplementary material for: Cancer cell responses to Hsp70 inhibitor JG-98: Comparison with Hsp90 inhibitors and finding synergistic drug combinations
Source: Sci Rep. 2018 Feb 14;8:3010. doi: 10.1038/s41598-017-14900-0 (PMC5813176; doi:10.1038/s41598-017-14900-0)
Supplement: Supplementary file 1 — Supplementary Information [file 41598_2017_14900_MOESM1_ESM.pdf]

Supplement

Cancer cell responses to Hsp70 inhibitor JG-98: Comparison with Hsp90 inhibitors and finding synergistic drug combinations.

Julia A. Yaglom, Yongmei Wang, Amy Li, Zhenghu Li, Stefano Monti, Ilya Alexandrov, Xiongbin Lu, Michael Y. Sherman

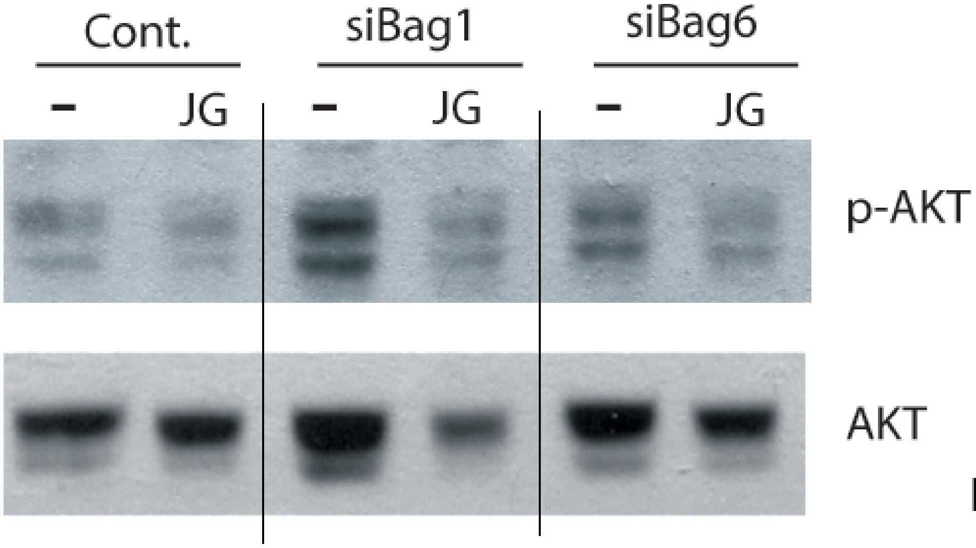

Fig. S1

Fig. S1. JG-98 downregulates AKT in Bag1- and Bag6-independent manner. MCF7 cells were transfected with either anti-Bag1 or anti-Bag6 siRNAs (see Materials and Methods) prior to treatment with 2μM of JG-98 for 36h. Levels of Akt and phAkt were determined in cell lysates by immunoblotting with corresponding antibody.

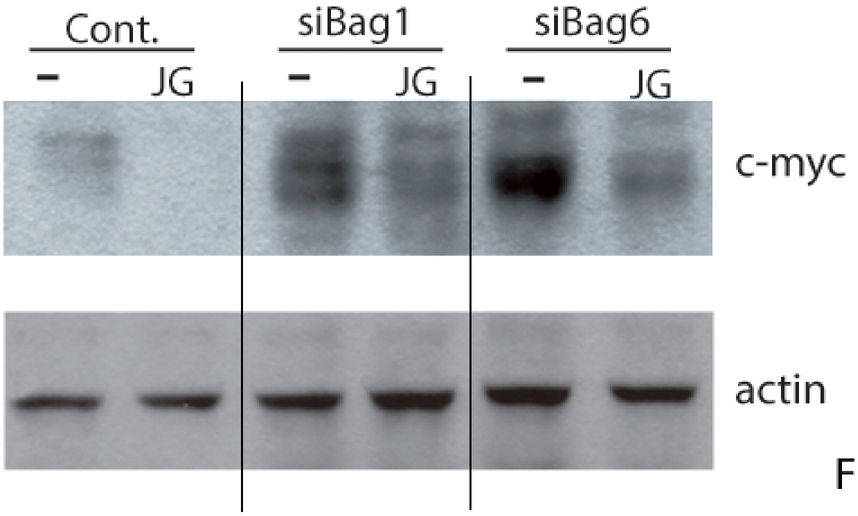

Fig. S2

Fig. S2. JG-98 downregulates c-myc in Bag1- and Bag6-independent manner. MCF7 cells were transfected with either anti-Bag1 or anti-Bag6 siRNAs as in Fig. S1 and treated with 2μM of JG-98 for 36h. Levels of c-myc were determined in cell lysates by immunoblotting with corresponding antibody.

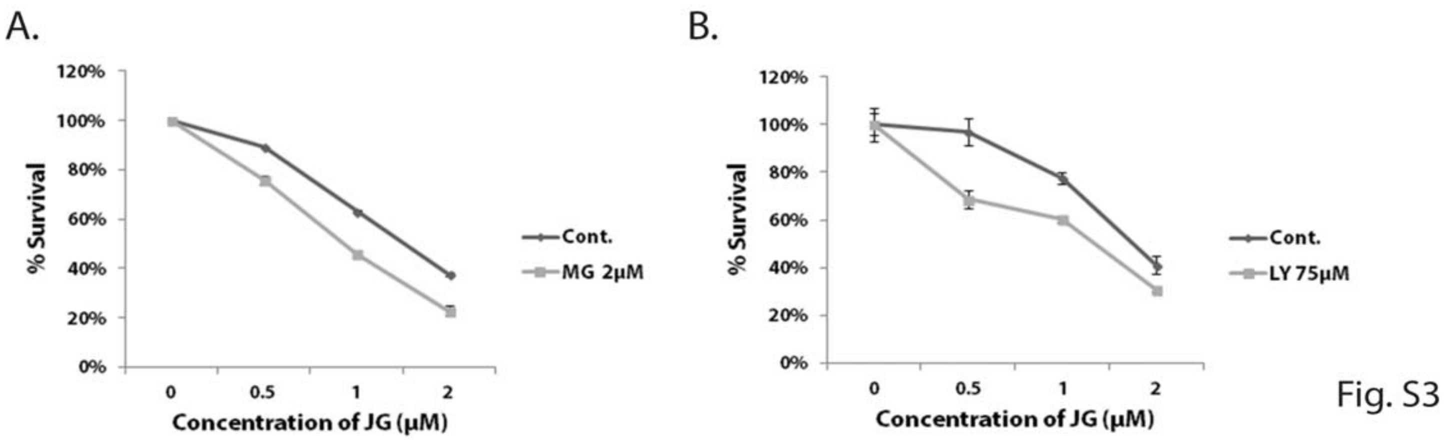

Fig. S3. Validation of effects of combinations of JG-98 with MG132 and LY294002 in BT474 cells. Sub-toxic concentrations of drugs were determined and effects of JG-98 either alone or in combination with these drugs were detyermined by MTT assay.

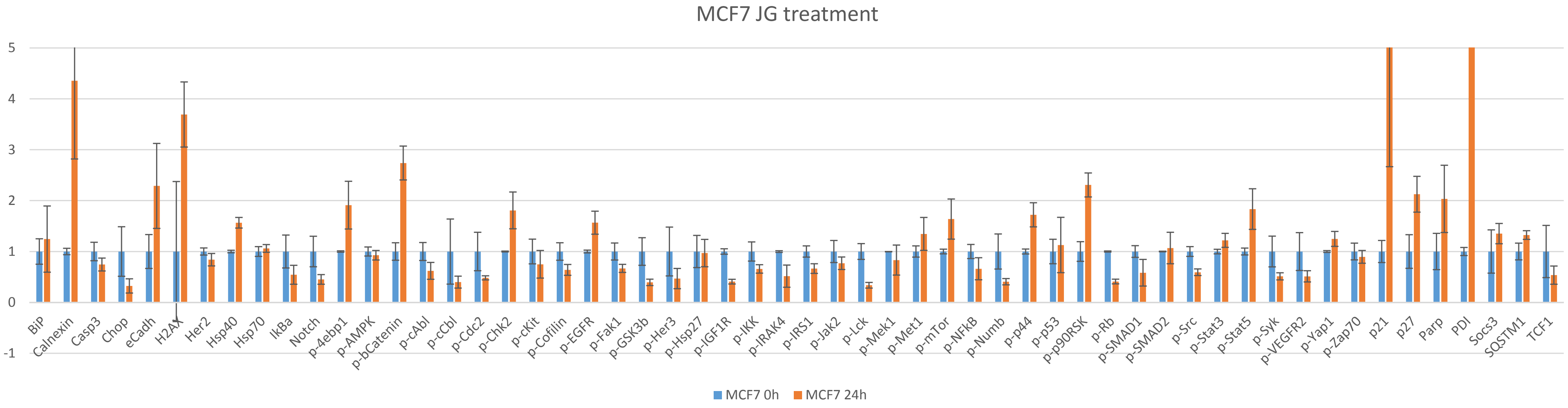
MCF7 JG treatment
5
4
3
2
1
0
-1
BiP
Calnexin
Casp3
Chop
eCadh
H2AX
Her2
Hsp40
Hsp70
IkBa
Notch
p-4ebp1
p-AMPK
p-bCatenin
p-cAbl
p-cCbl
p-Cdc2
p-Chk2
p-cKit
p-Cofilin
p-EGFR
p-Fak1
p-GSK3b
p-Her3
p-Hsp27
p-IGF1R
p-IKK
p-IRAK4
p-IRS1
p-Jak2
p-Lck
p-Mek1
p-Met1
p-mTor
p-NFkB
p-Numb
p-p44
p-p53
p-p90RSK
p-Rb
p-SMAD1
p-SMAD2
p-Src
p-Stat3
p-Stat5
p-Syk
p-VEGFR2
p-Yap1
p-Zap70
p21
p27
Parp
PDI
Socs3
SQSTM1
TCF1
MCF7 0h
MCF7 24h

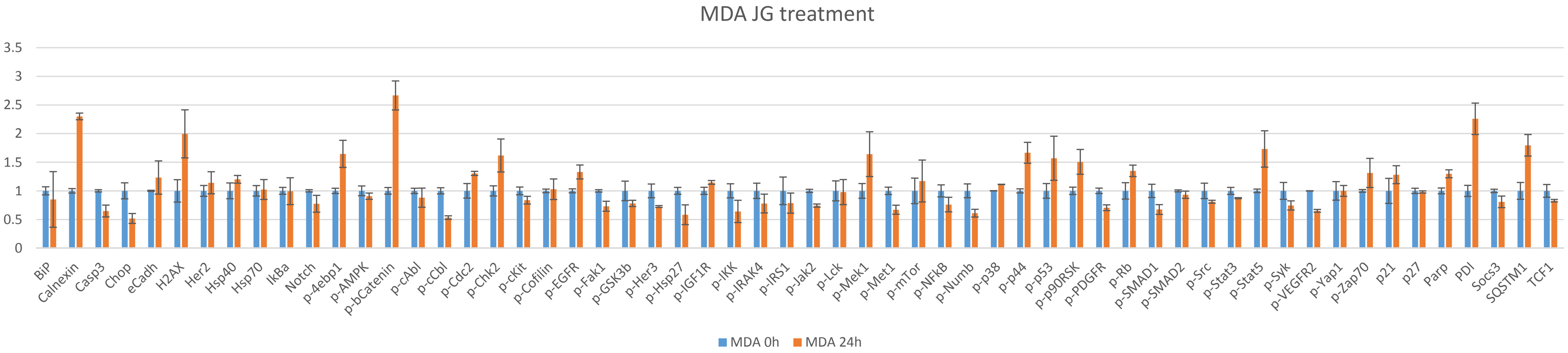
MDA JG treatment
3.5
3
2.5
2
1.5
1
0.5
0
BiP
Calnexin
Casp3
Chop
eCadh
H2AX
Her2
Hsp40
Hsp70
IkBa
Notch
p-4ebp1
p-AMPK
p-bCatenin
p-cAbl
p-cCbl
p-Cdc2
p-Chk2
p-cKit
p-Cofilin
p-EGFR
p-Fak1
p-GSK3b
p-Her3
p-Hsp27
p-IGF1R
p-IKK
p-IRAK4
p-IRS1
p-Jak2
p-Lck
p-Mek1
p-Met1
p-mTor
p-NFkB
p-Numb
p-p38
p-p44
p-p53
p-p90RSK
p-PDGFR
p-Rb
p-SMAD1
p-SMAD2
p-Src
p-Stat3
p-Stat5
p-Syk
p-VEGFR2
p-Yap1
p-Zap70
p21
p27
Parp
PDI
Socs3
SQSTM1
TCF1
MDA 0h
MDA 24h

Fig. S4

Fig. S4. Effect of JG-98 on multiple signaling proteins. The experiment was done using the iPAD platform by ActivSignal, Inc., see the text.

Table S1. Links to the Connectivity Map comparison of effects of JG-98 and tanespimycin. Links show drugs with connectivity to JG-98 and tanespimycin based on gene expression data in the database.

Table S1 cmap_report

Cell line	Pert name	Dose(µM)	url_hyperlink
HEPG2	tanespimycin	10	http://c3.lincscloud.org/~user165/mar23/my_analysis.sig_quest_tool.2016032314335591/query_result/tail.result_WTCS_POL001_HEPG2_24H_BRD-A61304759_10_n2000.html
HT29	tanespimycin	10	http://c3.lincscloud.org/~user165/mar23/my_analysis.sig_quest_tool.2016032314335591/query_result/tail.result_WTCS_POL001_HT29_24H_BRD-A61304759_10_n2000.html
JURKAT	tanespimycin	10	http://c3.lincscloud.org/~user165/mar23/my_analysis.sig_quest_tool.2016032314335591/query_result/tail.result_WTCS_POL001_JURKAT_24H_BRD-A61304759_10_n2000.html
MCF7	tanespimycin	10	http://c3.lincscloud.org/~user165/mar23/my_analysis.sig_quest_tool.2016032314335591/query_result/tail.result_WTCS_POL001_MCF7_24H_BRD-A61304759_10_n2000.html
PC3	tanespimycin	10	http://c3.lincscloud.org/~user165/mar23/my_analysis.sig_quest_tool.2016032314335591/query_result/tail.result_WTCS_POL001_PC3_24H_BRD-A61304759_10_n2000.html
HEPG2	tanespimycin	3.33333	http://c3.lincscloud.org/~user165/mar23/my_analysis.sig_quest_tool.2016032314335591/query_result/tail.result_WTCS_POL001_HEPG2_24H_BRD-A61304759_3.33333_n2000.html
HT29	tanespimycin	3.33333	http://c3.lincscloud.org/~user165/mar23/my_analysis.sig_quest_tool.2016032314335591/query_result/tail.result_WTCS_POL001_HT29_24H_BRD-A61304759_3.33333_n2000.html
JURKAT	tanespimycin	3.33333	http://c3.lincscloud.org/~user165/mar23/my_analysis.sig_quest_tool.2016032314335591/query_result/tail.result_WTCS_POL001_JURKAT_24H_BRD-A61304759_3.33333_n2000.html
MCF7	tanespimycin	3.33333	http://c3.lincscloud.org/~user165/mar23/my_analysis.sig_quest_tool.2016032314335591/query_result/tail.result_WTCS_POL001_MCF7_24H_BRD-A61304759_3.33333_n2000.html
PC3	tanespimycin	3.33333	http://c3.lincscloud.org/~user165/mar23/my_analysis.sig_quest_tool.2016032314335591/query_result/tail.result_WTCS_POL001_PC3_24H_BRD-A61304759_3.33333_n2000.html
HEPG2	tanespimycin	1.11111	http://c3.lincscloud.org/~user165/mar23/my_analysis.sig_quest_tool.2016032314335591/query_result/tail.result_WTCS_POL001_HEPG2_24H_BRD-A61304759_1.11111_n2000.html
HT29	tanespimycin	1.11111	http://c3.lincscloud.org/~user165/mar23/my_analysis.sig_quest_tool.2016032314335591/query_result/tail.result_WTCS_POL001_HT29_24H_BRD-A61304759_1.11111_n2000.html
JURKAT	tanespimycin	1.11111	http://c3.lincscloud.org/~user165/mar23/my_analysis.sig_quest_tool.2016032314335591/query_result/tail.result_WTCS_POL001_JURKAT_24H_BRD-A61304759_1.11111_n2000.html
MCF7	tanespimycin	1.11111	http://c3.lincscloud.org/~user165/mar23/my_analysis.sig_quest_tool.2016032314335591/query_result/tail.result_WTCS_POL001_MCF7_24H_BRD-A61304759_1.11111_n2000.html
PC3	tanespimycin	1.11111	http://c3.lincscloud.org/~user165/mar23/my_analysis.sig_quest_tool.2016032314335591/query_result/tail.result_WTCS_POL001_PC3_24H_BRD-A61304759_1.11111_n2000.html
HEPG2	tanespimycin	0.37037	http://c3.lincscloud.org/~user165/mar23/my_analysis.sig_quest_tool.2016

			032314335591/query_result/tail.result_WTCS_POL001_HEPG2_24H_BRD-A61304759_0.37037_n2000.html
HT29	tanespimycin	0.37037	http://c3.lincscloud.org/~user165/mar23/my_analysis.sig_quest_tool.2016032314335591/query_result/tail.result_WTCS_POL001_HT29_24H_BRD-A61304759_0.37037_n2000.html
JURKAT	tanespimycin	0.37037	http://c3.lincscloud.org/~user165/mar23/my_analysis.sig_quest_tool.2016032314335591/query_result/tail.result_WTCS_POL001_JURKAT_24H_BRD-A61304759_0.37037_n2000.html
MCF7	tanespimycin	0.37037	http://c3.lincscloud.org/~user165/mar23/my_analysis.sig_quest_tool.2016032314335591/query_result/tail.result_WTCS_POL001_MCF7_24H_BRD-A61304759_0.37037_n2000.html
PC3	tanespimycin	0.37037	http://c3.lincscloud.org/~user165/mar23/my_analysis.sig_quest_tool.2016032314335591/query_result/tail.result_WTCS_POL001_PC3_24H_BRD-A61304759_0.37037_n2000.html
HEPG2	tanespimycin	0.123457	http://c3.lincscloud.org/~user165/mar23/my_analysis.sig_quest_tool.2016032314335591/query_result/tail.result_WTCS_POL001_HEPG2_24H_BRD-A61304759_0.123457_n2000.html
HT29	tanespimycin	0.123457	http://c3.lincscloud.org/~user165/mar23/my_analysis.sig_quest_tool.2016032314335591/query_result/tail.result_WTCS_POL001_HT29_24H_BRD-A61304759_0.123457_n2000.html
JURKAT	tanespimycin	0.123457	http://c3.lincscloud.org/~user165/mar23/my_analysis.sig_quest_tool.2016032314335591/query_result/tail.result_WTCS_POL001_JURKAT_24H_BRD-A61304759_0.123457_n2000.html
MCF7	tanespimycin	0.123457	http://c3.lincscloud.org/~user165/mar23/my_analysis.sig_quest_tool.2016032314335591/query_result/tail.result_WTCS_POL001_MCF7_24H_BRD-A61304759_0.123457_n2000.html
PC3	tanespimycin	0.123457	http://c3.lincscloud.org/~user165/mar23/my_analysis.sig_quest_tool.2016032314335591/query_result/tail.result_WTCS_POL001_PC3_24H_BRD-A61304759_0.123457_n2000.html
HEPG2	tanespimycin	0.041152	http://c3.lincscloud.org/~user165/mar23/my_analysis.sig_quest_tool.2016032314335591/query_result/tail.result_WTCS_POL001_HEPG2_24H_BRD-A61304759_0.0411523_n2000.html
HT29	tanespimycin	0.041152	http://c3.lincscloud.org/~user165/mar23/my_analysis.sig_quest_tool.2016032314335591/query_result/tail.result_WTCS_POL001_HT29_24H_BRD-A61304759_0.0411523_n2000.html
JURKAT	tanespimycin	0.041152	http://c3.lincscloud.org/~user165/mar23/my_analysis.sig_quest_tool.2016032314335591/query_result/tail.result_WTCS_POL001_JURKAT_24H_BRD-A61304759_0.0411523_n2000.html
MCF7	tanespimycin	0.041152	http://c3.lincscloud.org/~user165/mar23/my_analysis.sig_quest_tool.2016032314335591/query_result/tail.result_WTCS_POL001_MCF7_24H_BRD-A61304759_0.0411523_n2000.html
PC3	tanespimycin	0.041152	http://c3.lincscloud.org/~user165/mar23/my_analysis.sig_quest_tool.2016032314335591/query_result/tail.result_WTCS_POL001_PC3_24H_BRD-A61304759_0.0411523_n2000.html
HEPG2	JG-98	5	http://c3.lincscloud.org/~user165/mar23/my_analysis.sig_quest_tool.2016032314335591/query_result/tail.result_WTCS_POL001_HEPG2_24H_BRD-U06877554_5_n2000.html
HT29	JG-98	5	http://c3.lincscloud.org/~user165/mar23/my_analysis.sig_quest_tool.2016032314335591/query_result/tail.result_WTCS_POL001_HT29_24H_BRD-U06877554_5_n2000.html
JURKAT	JG-98	5	http://c3.lincscloud.org/~user165/mar23/my_analysis.sig_quest_tool.2016032314335591/query_result/tail.result_WTCS_POL001_JURKAT_24H_BRD

			-U06877554_5_n2000.html
MCF7	JG-98	5	http://c3.lincscloud.org/~user165/mar23/my_analysis.sig_quest_tool.2016032314335591/query_result/tail.result_WTCS_POL001_MCF7_24H_BRD-U06877554_5_n2000.html
PC3	JG-98	5	http://c3.lincscloud.org/~user165/mar23/my_analysis.sig_quest_tool.2016032314335591/query_result/tail.result_WTCS_POL001_PC3_24H_BRD-U06877554_5_n2000.html
HEPG2	JG-98	1.66667	http://c3.lincscloud.org/~user165/mar23/my_analysis.sig_quest_tool.2016032314335591/query_result/tail.result_WTCS_POL001_HEPG2_24H_BRD-U06877554_1.66667_n2000.html
HT29	JG-98	1.66667	http://c3.lincscloud.org/~user165/mar23/my_analysis.sig_quest_tool.2016032314335591/query_result/tail.result_WTCS_POL001_HT29_24H_BRD-U06877554_1.66667_n2000.html
JURKAT	JG-98	1.66667	http://c3.lincscloud.org/~user165/mar23/my_analysis.sig_quest_tool.2016032314335591/query_result/tail.result_WTCS_POL001_JURKAT_24H_BRD-U06877554_1.66667_n2000.html
MCF7	JG-98	1.66667	http://c3.lincscloud.org/~user165/mar23/my_analysis.sig_quest_tool.2016032314335591/query_result/tail.result_WTCS_POL001_MCF7_24H_BRD-U06877554_1.66667_n2000.html
PC3	JG-98	1.66667	http://c3.lincscloud.org/~user165/mar23/my_analysis.sig_quest_tool.2016032314335591/query_result/tail.result_WTCS_POL001_PC3_24H_BRD-U06877554_1.66667_n2000.html
HEPG2	JG-98	0.555556	http://c3.lincscloud.org/~user165/mar23/my_analysis.sig_quest_tool.2016032314335591/query_result/tail.result_WTCS_POL001_HEPG2_24H_BRD-U06877554_0.555556_n2000.html
HT29	JG-98	0.555556	http://c3.lincscloud.org/~user165/mar23/my_analysis.sig_quest_tool.2016032314335591/query_result/tail.result_WTCS_POL001_HT29_24H_BRD-U06877554_0.555556_n2000.html
JURKAT	JG-98	0.555556	http://c3.lincscloud.org/~user165/mar23/my_analysis.sig_quest_tool.2016032314335591/query_result/tail.result_WTCS_POL001_JURKAT_24H_BRD-U06877554_0.555556_n2000.html
MCF7	JG-98	0.555556	http://c3.lincscloud.org/~user165/mar23/my_analysis.sig_quest_tool.2016032314335591/query_result/tail.result_WTCS_POL001_MCF7_24H_BRD-U06877554_0.555556_n2000.html
PC3	JG-98	0.555556	http://c3.lincscloud.org/~user165/mar23/my_analysis.sig_quest_tool.2016032314335591/query_result/tail.result_WTCS_POL001_PC3_24H_BRD-U06877554_0.555556_n2000.html
HEPG2	JG-98	0.185185	http://c3.lincscloud.org/~user165/mar23/my_analysis.sig_quest_tool.2016032314335591/query_result/tail.result_WTCS_POL001_HEPG2_24H_BRD-U06877554_0.185185_n2000.html
HT29	JG-98	0.185185	http://c3.lincscloud.org/~user165/mar23/my_analysis.sig_quest_tool.2016032314335591/query_result/tail.result_WTCS_POL001_HT29_24H_BRD-U06877554_0.185185_n2000.html
JURKAT	JG-98	0.185185	http://c3.lincscloud.org/~user165/mar23/my_analysis.sig_quest_tool.2016032314335591/query_result/tail.result_WTCS_POL001_JURKAT_24H_BRD-U06877554_0.185185_n2000.html
MCF7	JG-98	0.185185	http://c3.lincscloud.org/~user165/mar23/my_analysis.sig_quest_tool.2016032314335591/query_result/tail.result_WTCS_POL001_MCF7_24H_BRD-U06877554_0.185185_n2000.html
PC3	JG-98	0.185185	http://c3.lincscloud.org/~user165/mar23/my_analysis.sig_quest_tool.2016032314335591/query_result/tail.result_WTCS_POL001_PC3_24H_BRD-U06877554_0.185185_n2000.html

HEPG2	JG-98	0.061728	http://c3.lincscloud.org/~user165/mar23/my_analysis.sig_quest_tool.2016032314335591/query_result/tail.result_WTCS_POL001_HEPG2_24H_BRD-U06877554_0.0617284_n2000.html
HT29	JG-98	0.061728	http://c3.lincscloud.org/~user165/mar23/my_analysis.sig_quest_tool.2016032314335591/query_result/tail.result_WTCS_POL001_HT29_24H_BRD-U06877554_0.0617284_n2000.html
JURKAT	JG-98	0.061728	http://c3.lincscloud.org/~user165/mar23/my_analysis.sig_quest_tool.2016032314335591/query_result/tail.result_WTCS_POL001_JURKAT_24H_BRD-U06877554_0.0617284_n2000.html
MCF7	JG-98	0.061728	http://c3.lincscloud.org/~user165/mar23/my_analysis.sig_quest_tool.2016032314335591/query_result/tail.result_WTCS_POL001_MCF7_24H_BRD-U06877554_0.0617284_n2000.html
PC3	JG-98	0.061728	http://c3.lincscloud.org/~user165/mar23/my_analysis.sig_quest_tool.2016032314335591/query_result/tail.result_WTCS_POL001_PC3_24H_BRD-U06877554_0.0617284_n2000.html
HEPG2	JG-98	0.020576	http://c3.lincscloud.org/~user165/mar23/my_analysis.sig_quest_tool.2016032314335591/query_result/tail.result_WTCS_POL001_HEPG2_24H_BRD-U06877554_0.0205761_n2000.html
HT29	JG-98	0.020576	http://c3.lincscloud.org/~user165/mar23/my_analysis.sig_quest_tool.2016032314335591/query_result/tail.result_WTCS_POL001_HT29_24H_BRD-U06877554_0.0205761_n2000.html
JURKAT	JG-98	0.020576	http://c3.lincscloud.org/~user165/mar23/my_analysis.sig_quest_tool.2016032314335591/query_result/tail.result_WTCS_POL001_JURKAT_24H_BRD-U06877554_0.0205761_n2000.html
MCF7	JG-98	0.020576	http://c3.lincscloud.org/~user165/mar23/my_analysis.sig_quest_tool.2016032314335591/query_result/tail.result_WTCS_POL001_MCF7_24H_BRD-U06877554_0.0205761_n2000.html
PC3	JG-98	0.020576	http://c3.lincscloud.org/~user165/mar23/my_analysis.sig_quest_tool.2016032314335591/query_result/tail.result_WTCS_POL001_PC3_24H_BRD-U06877554_0.0205761_n2000.html

Table S2, S3. GSEA report showing pathways that are significantly upregulated (S2) or downregulated (S3) in response of MCF7 cells to JG- 98. MCF7 cells were treated with 2μM of JG-98 for 36 hours. RNA was isolated and submitted for microarray analysis. Microarray data were analyzed using the Broad Institute software package for gene set enrichment analysis (GSEA) in Boston University Center for Translational Research.

Table S2 downregulated pathways

NAME	SIZE	FDR q-val	FWER p-val
REACTOME_DNA_REPLICATION	182	0	0
REACTOME_CELL_CYCLE	383	0	0
REACTOME_CELL_CYCLE_MITOTIC	301	0	0
REACTOME_MITOTIC_M_M_G1_PHASES	162	0	0
REACTOME_CHROMOSOME_MAINTENANCE	108	0	0
REACTOME_S_PHASE	103	0	0
SGCGSSAAA_V$E2F1DP2_01	164	0	0
REACTOME_TELOMERE_MAINTENANCE	65	0	0
REACTOME_G1_S_TRANSITION	103	0	0
REACTOME_DEPOSITION_OF_NEW_CENPA_CONTAINING_NUCLEOSOMES_AT_THE_CENTROMERE	53	0	0
REACTOME_SYNTHESIS_OF_DNA	87	0	0
REACTOME_MITOTIC_G1_G1_S_PHASES	127	0	0
V$E2F1_Q6	229	0	0
V$E2F4DP2_01	230	0	0
V$E2F1DP2_01	230	0	0
V$E2F4DP1_01	235	0	0
REACTOME_PROCESSING_OF_CAPPED_INTRON_CONTAINING_PRE_MRNA	130	0	0
V$E2F1DP1_01	230	0	0
REACTOME_DNA_STRAND_ELONGATION	29	0	0
V$E2F_02	230	0	0
REACTOME_ACTIVATION_OF_ATR_IN_RESPONSE_TO_REPLICATION_STRESS	35	0	0
KEGG_DNA_REPLICATION	35	0	0
REACTOME_M_G1_TRANSITION	75	0	0
V$E2F1_Q3	240	0	0
V$E2F_Q6	227	0	0
REACTOME_MRNA_SPLICING	102	0	0
REACTOME_ACTIVATION_OF_THE_PRE_REPLICATIVE_COMPLEX	29	0	0
REACTOME_G2_M_CHECKPOINTS	41	0	0
REACTOME_MRNA_PROCESSING	148	0	0
V$E2F1DP1RB_01	225	0	0
V$E2F_Q4	229	0	0
REACTOME_MITOTIC_PROMETAPHASE	83	0	0
REACTOME_EXTENSION_OF_TELOMERES	26	0	0
REACTOME_CELL_CYCLE_CHECKPOINTS	109	0	0

V$E2F_Q3	222	0	0
REACTOME_E2F_MEDIATED_REGULATION_OF_DNA_REPLICATION	32	0	0
REACTOME_RNA_POL_I_PROMOTER_OPENING	47	0	0
REACTOME_ASSEMBLY_OF_THE_PRE_REPLICATIVE_COMPLEX	61	0	0
CHROMOSOME	120	0	0
V$E2F_03	236	0	0
V$E2F1_Q6_01	232	0	0
CHROMOSOMAL_PART	94	0	0
REACTOME_RNA_POL_I_RNA_POL_III_AND_MITOCHONDRIAL_TRANSCRIPTION	103	0	0
KEGG_OXIDATIVE_PHOSPHORYLATION	111	0	0
REACTOME_RNA_POL_I_TRANSCRIPTION	70	0	0
REACTOME_TRANSCRIPTION	186	0	0
REACTOME_TCA_CYCLE_AND_RESPIRATORY_ELECTRON_TRANSPORT	112	0	0
V$E2F_Q3_01	229	0	0
REACTOME_RESPIRATORY_ELECTRON_TRANSPORT_ATP_SYNTHESIS_BY_CHEMIOSMOTIC_COUPLING_AND_HEAT_PRODUCTION_BY_UNCOUPLING_PROTEINS_	76	0	0
V$E2F_Q4_01	232	0	0
RNA_SPLICING	88	0	0
KRCTCNNNNMANAGC_UNKNOWN	61	0	0
REACTOME_CHOLESTEROL_BIOSYNTHESIS	21	0	0
KEGG_SPLICEOSOME	121	0	0
V$E2F_Q6_01	234	0	0
BIOCARTA_MCM_PATHWAY	18	0	0
V$E2F1_Q4_01	222	0	0
REACTOME_ORC1_REMOVAL_FROM_CHROMATIN	63	0	0
REACTOME_LAGGING_STRAND_SYNTHESIS	18	0	0
MRNA_PROCESSING_GO_0006397	70	0	0
KEGG_PARKINSONS_DISEASE	107	0	0
REACTOME_TRANSPORT_OF_MATURE_TRANSCRIPT_TO_CYTOPLASM	52	0	0
M_PHASE_OF_MITOTIC_CELL_CYCLE	84	0	0
M_PHASE	113	0	0
KEGG_CELL_CYCLE	124	0	0
REACTOME_REGULATION_OF_MITOTIC_CELL_CYCLE	74	0	0
KEGG_SYSTEMIC_LUPUS_ERYTHEMATOSUS	113	0	0
REACTOME_G1_S_SPECIFIC_TRANSCRIPTION	17	0	0
REACTOME_MEIOTIC_RECOMBINATION	70	0	0
ORGANELLE_INNER_MEMBRANE	71	0	0
RNA_PROCESSING	165	0	0
RIBONUCLEOPROTEIN_COMPLEX	135	0	0
REACTOME_AMYLOIDS	67	0	0
REACTOME_MEIOSIS	98	0	0
CONDENSED_CHROMOSOME	31	0	0
REACTOME_RESPIRATORY_ELECTRON_TRANSPORT	61	0	0
REACTOME_MRNA_SPLICING_MINOR_PATHWAY	41	0	0
CELL_CYCLE_PROCESS	191	0	0

MITOSIS	81	0	0
MITOTIC_CELL_CYCLE	151	0	0
MITOCHONDRIAL_INNER_MEMBRANE	63	0	0
V$E2F_01	65	0	0
CELL_CYCLE_PHASE	168	0	0
REACTOME_METABOLISM_OF_NON_CODING_RNA	44	0	0
REACTOME_MEIOTIC_SYNAPSIS	62	0	0
REACTOME_CLEAVAGE_OF_GROWING_TRANSCRIPT_IN_THE_TERMINATION_REGION_	41	0	0
REPLICATION_FORK	18	0	0
REACTOME_DESTABILIZATION_OF_MRNA_BY_AUF1_HNRNP_D0	47	0	0
REACTOME_FORMATION_OF_TUBULIN_FOLDING_INTERMEDIATES_BY_CCT_TRIC	20	0	0
KEGG_STEROID_BIOSYNTHESIS	16	0	0
KEGG_GLYCOLYSIS_GLUCONEOGENESIS	60	0	0
REACTOME_CDT1_ASSOCIATION_WITH_THE_CDC6_ORC_ORIGIN_COMPLEX	52	0	0
REACTOME_PACKAGING_OF_TELOMERE_ENDS	39	0	0
NUCLEAR_CHROMOSOME	54	0	0
CHROMOSOMEPERICENTRIC_REGION	30	9.70E-06	0.001
RRNA_METABOLIC_PROCESS	16	9.60E-06	0.001
REACTOME_GLYCOLYSIS	26	9.50E-06	0.001
KEGG_OOCYTE_MEIOSIS	111	1.91E-05	0.002
REACTOME_CYCLIN_A_B1_ASSOCIATED_EVENTS_DURING_G2_M_TRANSITION	15	1.89E-05	0.002
REACTOME_APC_C_CDC20_MEDIATED_DEGRADATION_OF_MITOTIC_PROTEINS	62	1.87E-05	0.002
REACTOME_MRNA_3_END_PROCESSING	33	1.85E-05	0.002
CHROMATIN	34	1.83E-05	0.002
REACTOME_GLUCONEOGENESIS	30	4.60E-05	0.005
CONDENSED_NUCLEAR_CHROMOSOME	18	8.18E-05	0.009
CELL_CYCLE_GO_0007049	313	8.11E-05	0.009
SPINDLE	39	8.03E-05	0.009
REACTOME_CYCLIN_E_ASSOCIATED_EVENTS_DURING_G1_S_TRANSITION_	60	8.85E-05	0.01
REACTOME_G0_AND_EARLY_G1	23	8.77E-05	0.01
REACTOME_POST_CHAPERONIN_TUBULIN_FOLDING_PATHWAY	17	9.53E-05	0.011
REACTOME_DARPP_32_EVENTS	24	9.44E-05	0.011
MITOCHONDRIAL_PART	136	9.36E-05	0.011
DNA_REPLICATION	100	1.01E-04	0.012
MRNA_METABOLIC_PROCESS	81	1.00E-04	0.012
MITOCHONDRIAL_MEMBRANE	81	1.24E-04	0.015
REACTOME_APC_C_CDH1_MEDIATED_DEGRADATION_OF_CDC20_AND_OTHER_APC_C_CDH1_TARGETED_PROTEINS_IN_LATE_MITOSIS_EARLY_G1	61	1.23E-04	0.015
DNA_DEPENDENT_DNA_REPLICATION	55	1.70E-04	0.021
NUCLEOLAR_PART	18	1.77E-04	0.022
REACTOME_GLUCOSE_METABOLISM	63	1.75E-04	0.022
MITOTIC_SISTER_CHROMATID_SEGREGATION	15	1.82E-04	0.023
REACTOME_DNA_REPAIR	102	2.20E-04	0.028

RRNA_PROCESSING	15	2.34E-04	0.03
KEGG_PROGESTERONE_MEDIATED_OOCYTE_MATURATION	84	2.63E-04	0.034
SPLICEOSOME	48	2.83E-04	0.037
STEROID_BIOSYNTHETIC_PROCESS	23	2.88E-04	0.038
MITOCHONDRIAL_MEMBRANE_PART	49	3.01E-04	0.04
KEGG_TERPENOID_BACKBONE_BIOSYNTHESIS	15	3.43E-04	0.046
RIBONUCLEASE_ACTIVITY	25	3.70E-04	0.05
KEGG_BUTANOATE_METABOLISM	34	3.89E-04	0.053
REACTOME_HOST_INTERACTIONS_OF_HIV_FACTORS	116	4.58E-04	0.062
MITOCHONDRIAL_ENVELOPE	92	5.41E-04	0.074
NUCLEAR_CHROMOSOME_PART	34	5.80E-04	0.08
REACTOME_HIV_INFECTION	186	5.97E-04	0.082
REACTOME_RNA_POL_II_TRANSCRIPTION	97	6.00E-04	0.083
MITOCHONDRION	331	6.09E-04	0.085
REACTOME_HIV_LIFE_CYCLE	109	6.40E-04	0.089
KEGG_BASE_EXCISION_REPAIR	32	6.69E-04	0.094
KEGG_MISMATCH_REPAIR	23	6.64E-04	0.094
ORGANELLE_ENVELOPE	163	7.55E-04	0.108
SMALL_NUCLEAR_RIBONUCLEOPROTEIN_COMPLEX	22	7.84E-04	0.112
REACTOME_ABORTIVE_ELONGATION_OF_HIV1_TRANSCRIPT_IN_THE_ABSENCE_OF_TAT	23	8.39E-04	0.119
LIPID_BIOSYNTHETIC_PROCESS	95	8.46E-04	0.12
ENVELOPE	163	8.60E-04	0.123
REACTOME_SCFSKP2_MEDIATED_DEGRADATION_OF_P27_P21	51	8.92E-04	0.129
KEGG_PROTEASOME	42	0.001024	0.147
NUCLEOLUS	122	0.001095	0.158
REACTOME_TRANSPORT_OF_MATURE_MRNA_DERIVED_FROM_AN_INTRONLESS_TRANSCRIPT	32	0.001133	0.164
KEGG_PYRUVATE_METABOLISM	40	0.001138	0.165
REACTOME_METABOLISM_OF_CARBOHYDRATES	230	0.001137	0.166
CELL_CYCLE_CHECKPOINT_GO_0000075	48	0.001248	0.183
KEGG_PENTOSE_PHOSPHATE_PATHWAY	27	0.001284	0.19
REACTOME_CITRIC_ACID_CYCLE_TCA_CYCLE	18	0.001313	0.195
KEGG_PYRIMIDINE_METABOLISM	94	0.001311	0.196
REACTOME_TRANSCRIPTION_COUPLED_NER_TC_NER	41	0.001302	0.196
BIOCARTA_MPR_PATHWAY	34	0.001324	0.2
KEGG_VALINE_LEUCINE_AND_ISOLEUCINE_DEGRADATION	44	0.001358	0.205
KINETOCHORE	24	0.001368	0.208
DNA_METABOLIC_PROCESS	251	0.001383	0.211
SISTER_CHROMATID_SEGREGATION	16	0.001392	0.213
REACTOME_MITOTIC_G2_G2_M_PHASES	78	0.001402	0.216
REACTOME_GLOBAL_GENOMIC_NER_GG_NER	30	0.001393	0.216
KEGG_FATTY_ACID_METABOLISM	40	0.001384	0.216
CHROMATIN_BINDING	32	0.001376	0.216
REACTOME_KINESINS	24	0.00173	0.262
REACTOME_GLUCOSE_TRANSPORT	37	0.001737	0.265
KEGG_NUCLEOTIDE_EXCISION_REPAIR	42	0.001772	0.272
RIBOSOME_BIOGENESIS_AND_ASSEMBLY	18	0.001823	0.282
KEGG_HOMOLOGOUS_RECOMBINATION	28	0.002083	0.316
KTGGYRSGAA_UNKNOWN	75	0.002104	0.321

ALCOHOL_METABOLIC_PROCESS	86	0.002097	0.321
REACTOME_FORMATION_OF_THE_HIV1_EARLY_ELONGATION_COMPLEX	31	0.002157	0.329
KEGG_HUNTINGTONS_DISEASE	165	0.002155	0.331
REACTOME_NEP_NS2_INTERACTS_WITH_THE_CELLULAR_EXPORT_MACHINERY	26	0.002148	0.332
REACTOME_CDK_MEDIATED_PHOSPHORYLATION_AND_REMOVAL_OF_CDC6	44	0.002218	0.343
REACTOME_FATTY_ACYL_COA_BIOSYNTHESIS	18	0.002281	0.349
KEGG_PURINE_METABOLISM	155	0.002499	0.381
REACTOME_NUCLEOTIDE_EXCISION_REPAIR	46	0.002736	0.412

Table S3 upregulated pathways

NAME	FDR q-val	FWER p-val
REACTOME_UNFOLDED_PROTEIN_RESPONSE	0	0
REACTOME_BMAL1_CLOCK_NPAS2_ACTIVATES_CIRCADIAN_EXPRESSION	0	0
REACTOME_ACTIVATION_OF_CHAPERONE_GENES_BY_XBP1S	0	0
REACTOME_DIABETES_PATHWAYS	0	0
REACTOME_CIRCADIAN_CLOCK	0.013461	0.063
V$ATF6_01	0.02439	0.131
REACTOME_RORA_ACTIVATES_CIRCADIAN_EXPRESSION	0.02454	0.154
BIOCARTA_P53HYPOXIA_PATHWAY	0.022521	0.161
REACTOME_CIRCADIAN_REPRESSION_OF_EXPRESSION_BY_REV_ERBA	0.020951	0.168
REGULATION_OF_GTPASE_ACTIVITY	0.024243	0.209
TCTATGA,MIR-376A,MIR-376B	0.07349	0.546
BIOCARTA_PML_PATHWAY	0.06763	0.547
REACTOME_CYTOSOLIC_TRNA_AMINOACYLATION	0.068843	0.582
REACTOME_PERK_REGULATED_GENE_EXPRESSION	0.065437	0.586
ER_NUCLEAR_SIGNALING_PATHWAY	0.068671	0.627
KEGG_SNARE_INTERACTIONS_IN_VESICULAR_TRANSPORT	0.070436	0.662
GOLGI_VESICLE_TRANSPORT	0.068398	0.674
REACTOME_ANTIGEN_PRESENTATION_FOLDING_ASSEMBLY_AND_PEPTIDE_LOADING_OF_CLASS_I_MHC	0.067588	0.692
REACTOME_CELL_JUNCTION_ORGANIZATION	0.072851	0.738

Table S4. The list of genes that affect the response to JG-98 identified by the polled shRNA screen in MCF7 and MDA-MB231 cells.

Table S4

MCF7

**Up Genes**	**Down Genes**				
ACE	ABCB7	CYP1A2	HSD17B4	NR4A3	RUNX3
ACOT12	ABCC1	CYSLTR1	HSF2	NRG1	RUVBL2
ACVR1	ABCC2	DNAJA3	HSP90AB1	NT5C3	RXRA
ADH1C	ACADVL	DNAJB1	HSPB1	NUP214	SC5DL
AK1	ADCY8	DRD2	HSPG2	OCIAD1	SF1
APBB2	ADRA1A	DUSP4	HTR1F	OPRD1	SIN3A
ARF1	AGRN	DUSP6	HTR2B	OXTR	SLC26A2
ARFGAP3	ALDH4A1	EEF2	HTR2C	PAX8	SMARCA4
ATF2	AMPD2	EFTUD2	HTR3B	PCGF2	SNRPA1
ATG16L1	ANP32A	EIF1AX	HTR3C	PDHX	SNRPD2
ATP1A1	APOBEC3G	EIF2B5	HTR4	PGF	SORBS1
ATP5O	AQR	EIF2S2	HYAL2	PIK3CG	SP1
ATP6V1E2	ARFGEF2	EIF3A	IDH1	PIK4CA	STRAP
ATPGD1	ATF3	ELK4	IGFBP6	PITX2	TACR1
C1GALT1	ATG4A	EMR1	IL10RB	PLK1	TBXA2R
CACNA1B	ATP6AP1	EMR3	IL1R1	POLR2A	TCF7L1
CACNA1H	ATP6V1G1	ERCC3	IL22	POLR2B	TCOF1
CAMKK1	ATRX	ESRRA	IRF6	POLR2D	TEAD2
CAPN10	BAAT	FBXW7	IRF8	POLR2F	TFCP2
CD9	BAP1	FOXN1	JUP	POLR2I	TLE1
CDKL2	BMP10	FOXO1	KCNH7	POR	TNFSF4
CHEK1	BNIP3	FZD3	KIF11	PROCR	TNFSF8
CMA1	BPNT1	FZD4	KLRC4	PSMA1	TNIP1
COL4A3	C1ORF103	FZD7	KLRK1	PSMA2	UBE2L3
FOXK1	C7	GAD1	KPNB1	PSMA3	USP9X
GGT1	C9ORF47	GBX2	KREMEN1	PSMB2	VNN1
IFNA13	CASK	GCGR	LAT2	PSMB6	WARS2
IFNA16	CBR1	GGTL3	LDLR	PSMC2	WWTR1
INPP5D	CCK	GLP2R	LHX2	PSMD1	YY1
KCNJ3	CCL28	GNB2L1	LPHN2	PSMD2	
LHX8	CCL3L1	GPR109B	LRRK2	PSMD3	
LOC400652	CD58	GPR35	LY75	PSMD6	
LPO	CDA	GPR37	MAFK	PTGDR	
MAP3K3	CDC40	GPR68	MAML3	PTGER3	
MYH2	CDK5	GPR81	MAPK8IP3	RAN	
PA2G4	CDX4	GRM5	MBD1	RBX1	
PIGM	CHD8	GTF2E2	MC4R	RECQL	
PRKCB1	CLTC	GTF3C3	MCM2	RPA2	
RAC1	COL5A1	GZMB	MELK	RPL10	

RDH14	COL5A2	HNRNPC	MOG	RPL11
RENBP	CPO	HNRPK	MYL1	RPL14
SETD2	CRY1	HOXB4	NACA	RPL30
SMAD2	CSE1L	HOXB6	NCL	RPL6
SND1	CSF2RA	HOXD13	NEUROG3	RPS13
TNKS	CXCR3	HR	NMI	RTF1
XPC	CXCR6	HRH4	NR1D1	RUNX2

MDA-MB231

**Up Genes**	**Down Genes**			
ATP1A1	ABCC1	EIF2B5	PLA2G1B	SEC22B
IKZF3	ABCC2	EIF3A	PLK1	SERPINA10
	AKR1C2	EIF4EBP1	POLR2A	SHFM1
	ALG11	EREG	POLR2B	SKP1A
	ANAPC4	FCGR3B	POLR2D	SLAIN1
	AQR	FLT4	POLR2F	SMC6
	ARD1A	GAPDH	PON3	SMN1
	ATP1A4	GBF1	PPAP2B	SNCB
	ATP5G1	GBX2	PPP2R2A	SNRNP200
	ATP6AP1	GNA13	PRDX2	SNRPD2
	ATP6V0C	GPX4	PRKCE	SRP54
	ATP6V0D1	GSK3A	PRKRA	STAG2
	ATP6V1A	GSTA1	PRODH	STAR
	BAP1	HES6	PRPF19	STAT5A
	BCL2L1	HLA-E	PSMA1	STRAP
	BID	HNRNPC	PSMA2	THOC4
	BST2	HNRPK	PSMA3	TIMELESS
	CALM2	HSD17B1	PSMA6	TLN1
	CAPN5	IFNA17	PSMA7	TLR10
	CCL11	IFNG	PSMB2	TRAF3
	CCL13	IGF2R	PSMB4	TSC2
	CCL2	IL1RAP	PSMB6	TUBA1C
	CD226	IL2	PSMC1	UBE1
	CDC2L1	IL4R	PSMC2	UBE2I
	CDC40	ITGAV	PSMD1	UBE2L3
	CFLAR	KIF3A	PSMD11	UBE2V2
	CHD8	KPNB1	PSMD12	USP9X
	CHGB	LIAS	PSMD3	WARS2
	CLEC7A	LOC391656	PSMD8	XAB2
	COL8A1	LPHN3	PSMF1	XCL1
	COPS5	MCM4	RAB11A	YY1
	COQ7	MCM6	RAD21	ZFP42
	CPSF2	MTMR1	RAG1	
	CTNNA1	MYEF2	RAN	
	CXCR4	NACA	RARB	
	CYP21A2	NADSYN1	RBL2	

DARS	NARS	RBX1
DDHD1	NRAS	RFX4
DDX41	NT5C2	RPA2
DIDO1	NXT1	RPL30
DKK4	PAFAH1B2	RPL8
DLX3	PBEF1	RRM1
DPYSL2	PENK	RTF1
DSP	PFDN2	RUVBL2
EFTUD2	PIK3C3	SAP18
EIF1AX	PKN1	SCD5

Table S5. Top 40 connections that are predicted to sensitize cells to JG-98 according to analysis of the Connectivity Map.

Table S5 Top 40 Connections

	cmap_id	cmap_name	score_best2	Target
1	BRD-K13646352	midostaurin	99.822	TK
2	BRD-K35960502	niclosamide	99.486	
3	BRD-K74305673	IKK-2-inhibitor-V	99.147	
4	BRD-A58564983	selamectin	99.404	
5	BRD-A08003242	rhodomyrtoxin-b	99.134	
6	BRD-K01121114	BRD-K01121114	98.875	
7	BRD-K92241597	tosedostat	98.573	
8	BRD-K60623809	SU-11652	99.239	TK
9	BRD-K76840893	RS-17053	99.135	
10	BRD-K12184916	NVP-BEZ235	98.542	PI3K
11	BRD-K38477985	malonoben	98.732	
12	BRD-K67868012	PI-103	99.207	PI3K
13	BRD-K06750613	GSK-1059615	99.598	PI3K
14	BRD-K67075780	TGX-115	99.422	PI3K
15	BRD-K99582188	BRD-K99582188	97.737	
16	BRD-A34462049	tigecycline	99.135	
17	BRD-K68065987	MK-2206	98.773	Akt
18	BRD-A06352508	SB-218078	98.258	Chk1
19	BRD-K33272502	DG-041	97.765	
20	BRD-K86574132	BRD-K86574132	99.176	
21	BRD-K15616905	CCCP	98.324	
22	BRD-K55844427	BRD-K55844427	99.228	
23	BRD-A50737080	CGK-733	97.079	
24	BRD-K88677950	PD-198306	97.378	
25	BRD-K50836978	purvalanol-a	98.826	
26	BRD-K97365803	PI-828	99.261	PI3K
27	BRD-K23319301	BRD-K23319301	96.756	
28	BRD-A62809825	thapsigargin	97.405	
29	BRD-K63068307	ZSTK-474	99.11	PI3K
30	BRD-K67566344	KU-0063794	99.035	mTOR
31	BRD-K28934562	BRD-K28934562	97.185	
32	BRD-A59431241	COL-3	95.997	
33	BRD-K64642496	KO-143	96.465	
34	BRD-K73037408	KUC111774N	95.055	
35	BRD-K69932463	AZD-8055	96.998	mTOR
36	BRD-K40255344	tyrphostin-A9	97.587	TK
37	BRD-K59469039	AG-879	98.179	TK
38	BRD-A47829399	artesunate	97.551	
39	BRD-M64432851	sunitinib	99.152	TK
40	BRD-A11678676	wortmannin	97.616	mTOR
